# Efficient self-attention with smart pruning for sustainable large language models

**DOI:** 10.1038/s41598-025-92586-5

**Published:** 2025-03-24

**Authors:** Samir Brahim Belhaouari, Insaf Kraidia

**Affiliations:** 1 Division of Information and Computing Technology, College of Science and Engineering, Hamad Bin Khalifa University, Ar-Rayyan, Qatar; 2 Faculty of Information Technology, Department of Networks and Cybersecurity, Al-Ahliyya Amman University, Amman, Jordan

**Keywords:** Large Language Models (LLMs), Consumption, Computational Demands, Self-attention, Compression, Pruning, Computer science, Mathematics and computing

## Abstract

Large Language Models (LLMs) have revolutionized artificial intelligence by enabling multitasking across diverse fields. However, their high computational demands result in significant environmental impacts, particularly in terms of energy and water consumption. This paper addresses these issues by proposing an innovative compression approach to reducing LLM sizes. We focus on compressing the internal transformer layers, which are critical contributors to LLMs’ computational complexity. Our approach combines new mathematical and structural key methods for model compression. We begin by applying Forward Propagation Pruning (FPP) to compress the embedding and feed-forward layers, utilizing a weight freezing and zeroing technique for suspected unused parameters. This reduces the number of trainable parameters, accelerating the overall training process and enabling faster convergence. Second, the Weight Matrix Folding method is introduced to efficiently prune the self-attention layer matrices in a simple and efficient mathematical model. This method integrates Identical Row Compression (IRC) to optimize the compression of the Query and Key matrices, alongside Diagonal Weight Compression (DWC), which reformulates the Value matrix into a diagonal structure. Consequently, this technique significantly diminishes parameter variability across the three metrics, enhancing consistency and performance while simplifying complexity. The compression approach is evaluated on three language modeling datasets and eight widely used classification datasets, comparing it to various pruning methods. Our method successfully compresses transformer layers by 99% and linear layers by 70%, resulting in an overall model compression of around 70%, while maintaining nearly the same accuracy. Notably, with moderate compression rates of 20% to 40%, model performance not only remained stable but even improved. This leads to substantial reductions in memory usage and computational demands, making LLMs more resource-efficient and highlighting the potential to optimize them for a more sustainable AI future.

## Introduction

**Fig. 1:**
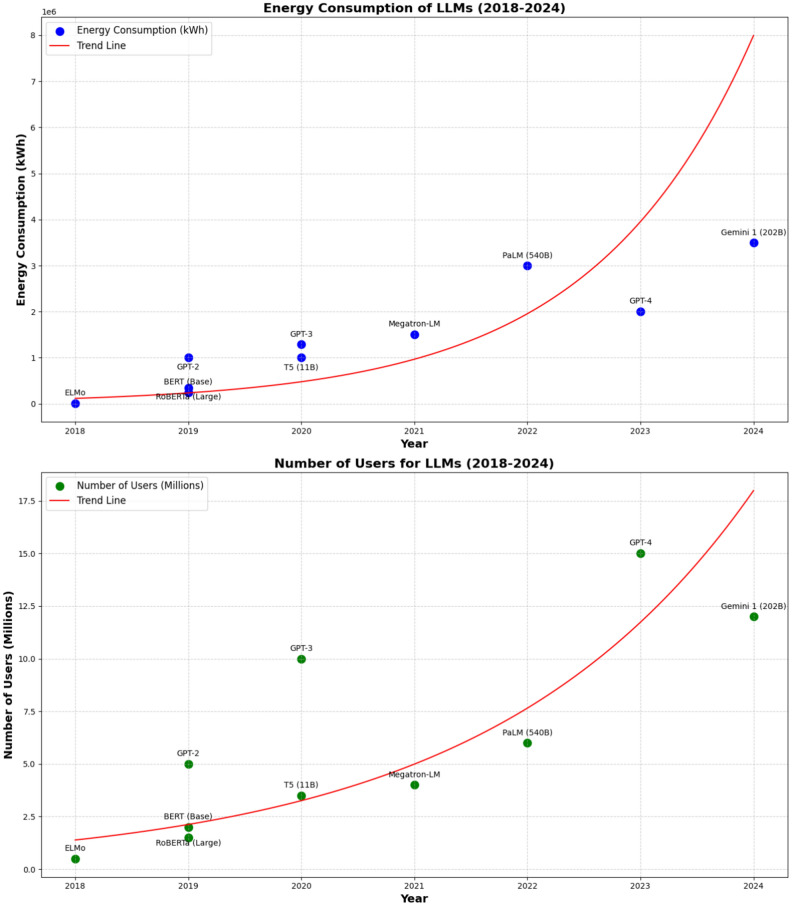
Energy consumption and user growth of LLMs (2018–2024). The red line represents the successive growth in LLM adoption and energy consumption across various domains. We report GPT-4 standing out for its significant user base of 15 millions users and Gemini reaching the highest energy consumption by 3.5 kWh in 2024.

Large Language Models, such as Generative Pre-trained Transformer 4 (GPT-4), represent a significant advancement in artificial intelligence, particularly their ability to handle multiple tasks across diverse domains. These models are designed to process vast amounts of text data, enabling them to perform various functions, from generating human-like text to answering complex questions, translating languages, text classification, and even assisting in creative endeavors^1^. The versatility of LLMs in multitasking across various fields has made them invaluable tools in industries ranging from content creation to customer support and research^2^. However, training and running large language models require significant computational resources, leading to high energy consumption^3^. The environmental impact of scaling LLMs is significant, with rising carbon and water emissions. Figure 1 shows the increasing energy consumption of major LLMs from 2018 to 2024, driven by growing complexity and user demand, underscoring the tension between innovation and sustainability^4^.

LLMs require immense computational resources, leading to significant energy consumption and environmental impact^5^. For example, training GPT-3 emitted carbon equivalent to driving 112 gasoline cars for a year^6^, while GPT-4’s training consumed about 7.5 MWh of energy—similar to the annual energy use of 700 U.S. households. Furthermore, maintaining these models and processing prompts add to their carbon footprint. LLMs also have a substantial water footprint, with ChatGPT requiring about 500 ml of water for every 20–50 prompts^7^ and around 700,000 liters^8^ during training. These models are housed in global data centers, consuming energy and water for cooling.

As the threats posed by climate change become increasingly critical, the need for urgent action grows ever more pressing, new technologies must meet the global sustainability goals. While there are ways in which LLMs and other AI technologies can contribute positively to sustainable development, these benefits can easily be outweighed by the immense energy and water costs they bring. The growing computational power required to train and deploy these models exacerbates the strain on global resources, necessitating a revaluation of how these models are developed and maintained. Optimization techniques, such as model pruning^9,10^ and efficient resource management, are crucial in addressing these challenges^11^. By reducing models’ size without compromising performance, we can mitigate the environmental impact, reduce operational costs, and make AI more sustainable in the long run. This research proposes a novel approach to pruning large language models by applying new mathematical and structural compression approaches to the architecture throughout the training process. These approaches involve strategically reducing model size while preserving or improving performance, leading to more efficient LLMs in terms of memory usage and computational cost. Our contributions can be summarized as follows:

- A new cumulative Adam technique is proposed to optimize deep neural networks by enhancing the Adam algorithm with a focus on the overall trend in loss reduction, improving the model’s learning.
- An effective mathematical compression method for the self-attention mechanism is developed, enhancing performance and optimizing efficiency.
- A novel structural Forward Propagation Pruning method is introduced to compress FeedForward and embedding representations in LLMs.
- A sequential combination approach for compressing LLM layers is presented, achieving a 70% overall compression rate, and evaluated on three language modeling tasks and eight text classification datasets using performance and inference metrics.

The remainder of the paper is structured as follows: Sect. “Related work” reviews recent trends in LLM pruning, Sect. “Background” provides background on LLM architectures, Sect. “Methodology” outlines our proposed methodology, Sect. “Training mechanism” provides our training mechanism, and Sect. “Results and discussion” presents an overview of the performance of our approach. Finally, Sect. “Conclusions” concludes the paper by discussing the limitations of our work and potential directions for future research.

## Related work

The compression of language models has gained significant attention, leading to the development of various methods, including network pruning^12^, knowledge distillation, and quantization^13^. LLM-Pruner^14^ and FLAP^15^ aim to reduce network width by pruning coupled structures. Sheared-LLaMA^16^ takes a more comprehensive approach, reducing network width and depth by removing entire layers. While methods that address both width and depth aspects exist^17,18^, there is still a need for in-depth analysis comparing the impact of these factors on LLM inference efficiency. Applying traditional pruning techniques to LLMs presents unique challenges due to their vast number of parameters and substantial computational requirements^19^. Pruning methods for LLMs can be broadly categorized into unstructured and structured approaches.

Structured pruning methods^20^ focus on removing entire groups of parameters, maintaining dense weight matrices, and improving hardware efficiency. Techniques such as LLM-Pruner^14^ and LoRAPrune^11^ emphasize efficient deployment and inference acceleration. Sheared-LLaMA^16^ aims to prune models to a target architecture and train them dynamically. The work of Tao introduces a novel compression technique called QuantGPT. This method focused on quantization and module adaptation, allowing for systematic compression while maintaining the integrity of the model’s architecture and performance^25^. LightPAFF focuses on transferring knowledge from a more prominent (teacher) model to a smaller (student) model. This process inherently involves structuring the model to retain important features learned by the teacher rather than simply removing individual weights, as seen in unstructured pruning^21^. Unstructured pruning methods^22^ target individual weights, maintaining performance. Notable examples include SparseGPT^23^, which employs sophisticated weight updates and pruning without retraining. Edalati et al. developed KnGPT2 for compressing the linear mappings of the GPT-2 model, focusing on reducing the number of parameters flexibly without drastically altering the overall architecture. This technique allows for representing weight matrices in a more compact form while maintaining performance, which aligns with the characteristics of unstructured pruning^24^.

Structure pruning typically removes entire groups of parameters, such as whole neurons, channels, or even layers, which can limit its flexibility^25^. Furthermore, this method can achieve a different level of sparsity than unstructured pruning, limiting its ability to compress models efficiently without retraining or redesigning the model architecture^26^. On the other hand, unstructured pruning operates at the level of individual weights in a neural network, meaning it can remove any specific weight, regardless of its position^27^. This allows for more fine-grained control over which weights to prune. This can target only the least essential weights, leading to a more refined reduction in model size while retaining critical model capacity^28^. It can achieve higher sparsity levels without compromising model performance because it is not constrained by the rigid structures to which structured pruning must adhere.

Despite preserving model performance, unstructured pruning often results in sparse weight matrices, which may not fully utilize hardware efficiency, as specialized hardware (such as GPUs) is typically optimized for dense matrix operations. To address the limitations of unstructured pruning in efficiently utilizing hardware resources, we propose a novel approach that combines Identical Row Compression, Diagonal Weight Compression for self-attention mechanisms, and linear weight freezing for the feed-forward (FF) and embedding layers. By integrating these methods, we create a model that retains performance and enhances computational efficiency. The combination of IRC and DWC allows for a reduction in the number of active parameters while maintaining a structure that can be efficiently processed on hardware optimized for dense operations. Meanwhile, linear weight freezing stabilizes the training process and reduces computational overhead in the feed-forward and embedding layers. Overall, this multi-faceted approach effectively addresses the challenges associated with unstructured pruning while enhancing hardware efficiency and model performance.

## Background

The architecture of LLMs is primarily based on transformers, which rely on self-attention mechanisms to capture relationships between words in a sequence. Unlike traditional models that process data sequentially, transformers handle input data in parallel, making them highly efficient for processing long text sequences. Essential layers in LLMs include:

### Embedding layer

The embedding layer converts input tokens into continuous dense vectors, allowing the model to work with numerical representations. It also includes positional encodings to help the transformer recognize the order of tokens in the sequence, which is essential for capturing relationships in language^29^.

### Multi-Head self-attention layer

The multi-head self-attention layer is a core component of the transformer architecture. It is responsible for capturing relationships between tokens in a sequence by computing attention scores^14^. It enables the model to focus on different input parts simultaneously, critical to its efficiency and performance.

For each token in the input sequence, three distinct matrices are generated: Query (Q), Key (K), and Value (V). These matrices are learned projections of the token embeddings. The Query matrix represents the token for which attention is being calculated, the Key matrix represents other tokens in the sequence that attention is directed towards, and the Value matrix contains the actual information of each token^30^. The attention mechanism then weighs the Value matrix based on its importance, which is determined by comparing the Query and Key matrices.

Attention is computed by taking the dot product of the Q with the K and then dividing it by the square root of the dimensionality of the Key to scale the result. A $softmax$ function is applied to the attention scores to normalize them, turning these scores into probabilities that sum to 1. These probabilities indicate how much focus the model should place on each token in the sequence relative to the current token, as illustrated in Eq. (1):

1 $$\text{Attention}\left(\text{Q},\text{K},\text{V}\right)=\text{softmax}\left(\frac{Q{K}^{T}}{\sqrt{{d}_{k}}}\right)\text{V}$$

where:

$$Q=X{W}_{Q}$$

$$K=X{W}_{K}$$

$$V=X{W}_{V}$$

here, $X,$ $\text{X}\in {\text{R}}^{n\times d},$ represents the input sequence of length $n$ and dimensionality $d$. The weight matrices ${W}_{Q},{W}_{K}\in {R}^{d\times {d}_{Q}}$ and ${W}_{V}\in {R}^{d\times {d}_{V}}$ are learned parameters responsible for creating the Query, Key, and Value matrices from the input embeddings. In this context, ${d}_{Q}$ denotes the dimensionality of the Keys and Queries, while ${d}_{V}$ denotes the dimensionality of the Values. The $softmax$ function ensures that the attention scores are normalized across all tokens, allowing the model to weigh the importance of each token in the sequence appropriately.

For the Multi-Head Mechanism, instead of calculating a single attention score for each token pair, the model uses multiple attention heads. Each attention head independently computes attention, and their results are concatenated and then linearly transformed into the final output, as illustrated in Eq. (2):

2 $$\text{MultiHead}\left(\text{Q},\text{K},\text{V}\right)=\text{Concat}\left({head}_{1}\dots \dots {head}_{h}\right){W}^{O}$$

where ${W}^{O}$ is a learned weight matrix applied to the concatenated output of the heads.

### Feedforward layer (FF)

The feedforward layer applies a nonlinear transformation to the output of the self-attention layer. It processes each token independently through a series of fully connected layers, enhancing the model’s capacity to learn complex patterns from the attention mechanism^31^. After each feedforward layer, a normalization layer is applied to stabilize training by normalizing inputs and improving convergence, working in conjunction with residual connections that help preserve information from earlier layers and enhance gradient flow, enabling more effective training of deeper models. Finally, the output layer maps the final token representations to predictions, often using $SoftMax$ for tasks like text generation.

## Methodology

LLMs, being deep neural networks, have several layers, including self-attention mechanisms, feedforward networks, and embedding layers, all of which require significant amounts of memory and computation. The self-attention mechanism, for example, involves the calculation of Query, Key, and Value matrices. These matrices grow in size as the model scales, leading to increased memory and processing demands. By compressing the model weights, whether through techniques such as pruning^12,22^, quantization^32^, or low-rank approximations^11^, we can reduce the size of these matrices and consequently lower both memory and computation requirements. This is particularly important when deploying models in resource-constrained environments where energy consumption, memory availability, and inference speed are crucial factors. However, Direct compression methods, often lead to reduced model accuracy because they can disrupt the model’s ability to maintain performance. These techniques simplify the network by removing or approximating weights, which may compromise the model’s capacity to capture intricate patterns in data^33^. Consequently, while these methods reduce memory usage and computational demands, they can also result in a decrease in model accuracy and stability, making it challenging to balance efficiency with performance.

**Fig. 2:**
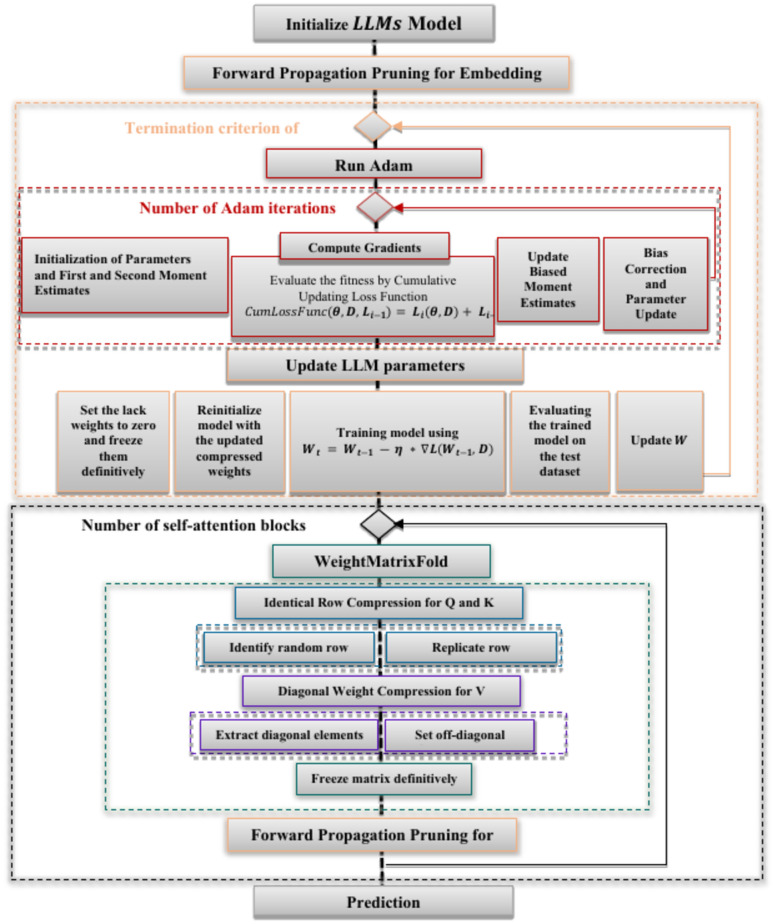
Flowchart of the proposed compression methodology. Each process is represented by different flow colors. FFP (orange flows) is applied to the embedding layer and each self-attention layer, incorporating cumulative Adam optimization (red flow). WeightMatrixFold (green flow) is applied to each feedforward layer under the self-attention layer, including Identical Row Compression (blue flows) and DWC (purple flow) methods.

Our new compression approach leverages two key strategies: Sequential and Recursive. As shown in Fig. 2, the Sequential strategy involves compressing the model layer by layer in a specific order. We begin by compressing the embedding layer (with ${W}_{0}$ representing the embedding layer’s weights) using the Forward Propagation Pruning method (orange flows), incorporating cumulative strategies based on the Adam optimizer (red flows). The optimized weights from this layer are then used to compress the self-attention block (black flow). For the self-attention layer, we apply the WeightMatrixFold technique (green flows), which utilizes Identical Row Compression for the Q and K matrices (blue flows) and Diagonal Weight Compression for the V matrix (purple flows). Finally, we apply the Forward Propagation Pruning method again to compress the corresponding feedforward layer before moving on to the next self-attention block. This ensures that each layer benefits from the optimizations of the previous layers. This method ensures a more efficient compression process, where each layer progressively improves based on the compression applied to earlier layers. Let $C({W}_{i})$ denote the compression function applied to layer $i$. For each layer $i$, the compression process can be formulated by Eq. (3). This equation states that the compressed weight matrix $\acute{W}_{i}$ of layer $i$ is the result of applying the compression function $C$ to the original weight matrix ${W}_{i}$, conditioned on the compressed weight matrices from the previous layers $\acute{W}_{i - 1} ,\acute{W}_{i - 2} ,\acute{W}_{i - 3} ,...\acute{W}_{0}$.

3 $$\acute{W}_{i - 1} = C(\left. {W_{i} } \right|\acute{W}_{i - 1} ,\acute{W}_{i - 2} ,\acute{W}_{i - 3} ,...\acute{W})_{0}$$

**Fig. 3:**
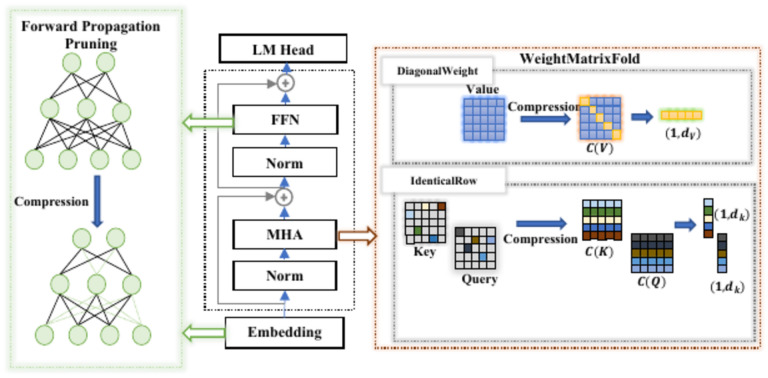
The Forward Propagation Pruning and WeightMatrixFold methods within a transformer-based architecture. In the Embedding and Feed-Forward Network (FFN) sub-layers, we prune connections between neurons. In the Multi-Head Attention (MHA) sub-layer, we prune connections between the input tokens and the attention scores or output.

Recursive strategy, on the other hand, focuses on gradually compressing the model, as illustrated in Algorithm 1. This strategy compresses a weight matrix iteratively, starting with a low compression rate ${r}_{min}$ and increasing it gradually ${r}_{t}$=${r}_{t-1}+\Delta r$. After each compression, the model’s performance is evaluated, $P(\acute{W}_{t} )$ and if the performance exceeds a predefined threshold $\epsilon$, the optimal compressed weights $(\acute{W}_{T} )$ and $\epsilon$ are updated. The process continues for a set number of iterations or until the performance threshold is met. This incremental approach helps maintain model accuracy during compression, allowing for fine-tuning and preventing significant performance degradation. Together, these strategies optimize model size and efficiency without compromising performance, achieving a more balanced trade-off between compression and accuracy. Figure 3 illustrates the impact of these methods on transformer architectures, demonstrating how the layers are affected. In the following section, we explain each method and its effects on computational efficiency and memory usage.

**Algorithm 1:**
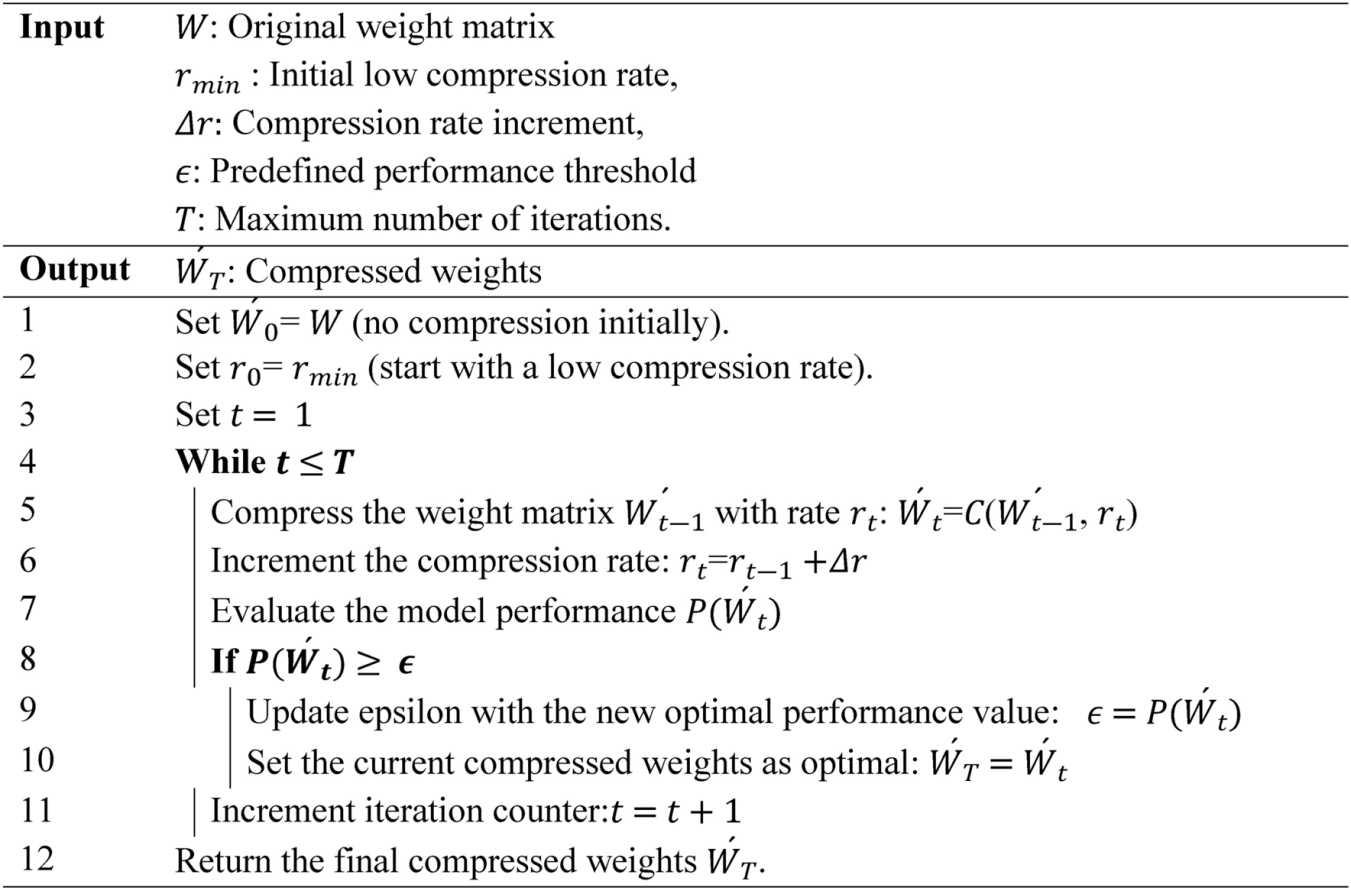
Recursive compression algorithm.

### Weight matrix fold (WMF)

Self-attention is a mechanism that allows a model to weigh the significance of different parts of an input sequence when processing data. This concept has gained prominence in natural language processing (NLP) and is a key component of transformer architectures, which have revolutionized tasks like language translation, text generation, and more. Our new simple mathematical model will enhance the idea behind self-attention to achieve better performance with fewer parameters. Focusing on compressing the self-attention mechanism in transformers, particularly the Q, K, and V matrices, is strategic because self-attention is one of the most computationally expensive parts of LLM. It scales quadratically with input length, meaning that as input sequences grow, self-attention’s computational and memory demands increase. By optimizing self-attention first, we target the biggest bottleneck in transformer models, achieving significant efficiency gains without heavily impacting performance, making it a priority in model compression efforts.

#### Identical row compression for Q and K(IRC)

One promising direction is compressing the Q and K matrices, which play a central role in attention computation. The primary idea behind this method is to reduce the redundancy in the $Q$ and $K$ matrices by leveraging row repetition. In this approach, the idea is to drastically simplify the $Q$ and $K$ matrices by assigning a single weight per token, rather than a full vector of weights. Instead of computing a separate query (Q) or key (K) vector for each token, each token is associated with one scalar weight. This scalar weight is then used for all positions in the matrix, which means that each row in the $Q$ and $K$ matrices become identical for a given token. By doing this, the attention mechanism still operates, but the computational complexity is significantly reduced since the matrix multiplication operations become far simpler. This method focuses on capturing the most critical relationship between tokens, at the cost of some expressiveness in the attention mechanism, but it leads to a highly compressed model that is computationally efficient and less memory intensive. Firstly, we initialize a random matrix (${W}_{Q}$​ and ${W}_{K}$​) and select a single row at random. This selected row is replicated across all matrix rows, reducing the matrix’s variability and the number of unique computations performed during self-attention. The compressed form of ${W}_{K}$ and ${W}_{Q}$ is updated by ${{\acute{W}}_{Q}}$ and ${{\acute{W}}_{K}}$ in Eq. (4) and Eq. (5).

4 $$\acute{W}_{Q} = (q_{1} ,q_{2} ,...,q_{d} )^{T} 1_{{1 \times d_{Q} }}$$

5 $${{\acute{W}}_{K}}={\left({k}_{1},{k}_{2},\dots ,{k}_{d}\right)}^{T}{1}_{1\times {d}_{Q}},$$

where ${1}_{1\times {d}_{Q}}$ is a unit vector of size one by ${d}_{Q}$, while $q,k\in {R}^{d}$ are vectors corresponding to the weights of the compressed $Q$ and $K$ matrices.

Our method introduces structured sparsity and controlled redundancy, compressing the matrix without costly decompositions like pruning^34^. This transformation reduces the variability in the matrix and the number of unique dot products calculated during attention. Notably, the compression method is simple to implement and does not introduce additional matrix factorization steps, which makes it computationally efficient. The new forms of the ${{\acute{W}}_{Q}}$ and ${{\acute{W}}_{K}}$ matrices are described by Eq. (6) and (7):

6 $${{\acute{W}}_{Q}}=\left[\begin{array}{c}\begin{array}{c}{q}_{1}\\ {q}_{2}\\ .\end{array}\\ .\\ {q}_{d}\end{array}\right]\left[1 1\dots 1\right]=\left[\begin{array}{cc}\begin{array}{cc}{q}_{1}& {q}_{1}\\ {q}_{2}& {q}_{2}\end{array}& \begin{array}{cc}\dots & {q}_{1}\\ \cdots & {q}_{2}\end{array}\\ \begin{array}{cc}\vdots & \ddots \\ {q}_{d}& {q}_{d}\end{array}& \begin{array}{cc}\dots & \vdots \\ \dots & {q}_{d}\end{array}\end{array}\right]$$

7 $${{\acute{W}}_{K}}=\left[\begin{array}{c}\begin{array}{c}{k}_{1}\\ {k}_{2}\\ .\end{array}\\ .\\ {k}_{d}\end{array}\right]\left[1 1\dots 1\right]=\left[\begin{array}{cc}\begin{array}{cc}{k}_{1}& {k}_{1}\\ {k}_{2}& {k}_{2}\end{array}& \begin{array}{cc}\dots & {k}_{1}\\ \cdots & {k}_{2}\end{array}\\ \begin{array}{cc}\vdots & \ddots \\ {k}_{d}& {k}_{d}\end{array}& \begin{array}{cc}\dots & \vdots \\ \dots & {k}_{d}\end{array}\end{array}\right]$$

Next, for the input vector $$X=\left[\begin{array}{c}{x}_{1}\\ \begin{array}{c}{x}_{2}\\ \vdots \end{array}\\ {x}_{n}\end{array}\right], {\text{where}\,x}_{i}=[{x}_{i1},{x}_{i2},\dots ,{x}_{iq}]\in {R}^{d}$$, the self-attention score $Q{K}^{T}$ can be expressed using the product of the transformed query and key matrices as shown in Eq. (8):

8 $$Q{K}^{T}={{X}{\acute{W}}_{Q}}{\left(X. {{\acute{W}}_{K}}\right)}^{T}$$

This formulation computes the attention scores efficiently by leveraging the compressed representations of the queries and keys. Expanding this gives Eq. (9):

$${X}{\acute{W}_{Q}}{\left(X. {{\acute{W}}_{K}}\right)}^{T}=\left[\begin{array}{c}{x}_{1}\\ \begin{array}{c}{x}_{2}\\ \vdots \end{array}\\ {x}_{n}\end{array}\right]\left[\begin{array}{c}\begin{array}{c}{q}_{1}\\ {q}_{2}\\ .\end{array}\\ .\\ {q}_{d}\end{array}\right]\left[1 1 . . . 1\right]\left[\begin{array}{c}1\\ \begin{array}{c}1\\ \vdots \end{array}\\ 1\end{array}\right]\left[\begin{array}{cc}\begin{array}{cc}{k}_{1}& {k}_{2}\end{array}& \begin{array}{cc}\dots & {k}_{d}\end{array}\end{array}\right]{\left[\begin{array}{c}{x}_{1}\\ \begin{array}{c}{x}_{2}\\ \vdots \end{array}\\ {x}_{n}\end{array}\right]}^{T}$$

The product of ${1}_{1\times {d}_{Q}}$ and ${1}_{{d}_{Q}\times 1}$ is simply the scalar ${d}_{Q}$. Therefore, we can simplify the equation to:

$${X}{\acute{W}_{Q}}{\left(X. {{\acute{W}}_{K}}\right)}^{T}={d}_{Q}\left[\begin{array}{c}{x}_{1}\\ \begin{array}{c}{x}_{2}\\ \vdots \end{array}\\ {x}_{n}\end{array}\right]\left[\begin{array}{ccc}\begin{array}{cc}{q}_{1}{k}_{1}& {q}_{1}{k}_{2}\\ {q}_{2}{k}_{1}& {q}_{2}{k}_{2}\end{array}& \begin{array}{cc}{q}_{1}{k}_{3}& {q}_{1}{k}_{4}\\ {q}_{2}{k}_{3}& {q}_{2}{k}_{4}\end{array}& \begin{array}{cc}\dots & {q}_{1}{k}_{d}\\ \dots & {q}_{2}{k}_{d}\end{array}\\ \begin{array}{cc}{q}_{3}{k}_{1}& {q}_{3}{k}_{2}\\ \vdots & \dots \end{array}& \begin{array}{cc}{q}_{3}{k}_{3}& {q}_{3}{k}_{4}\\ \dots & \dots \end{array}& \begin{array}{cc}\dots & {q}_{3}{k}_{d}\\ \dots & \vdots \end{array}\\ \begin{array}{cc}\vdots & \vdots \\ {q}_{n}{k}_{1}& {q}_{n}{k}_{2}\end{array}& \begin{array}{cc}\vdots & \vdots \\ {q}_{n}{k}_{3}& {q}_{n}{k}_{4}\end{array}& \begin{array}{cc}\vdots & \vdots \\ \dots & {q}_{n}{k}_{d}\end{array}\end{array}\right]\left[\begin{array}{cc}\begin{array}{cc}{x}_{1}& {x}_{2}\end{array}& \begin{array}{cc}\dots & {x}_{n}\end{array}\end{array}\right]$$

9 $${XW}_{Q}{\left(X. {{\acute{W}}_{K}}\right)}^{T} ={d}_{Q}A,$$

where the matrix $A$ results from the element-wise operations between the compressed vectors. Each element ${a}_{ij}$ of the matrix $A$ is defined by Eq. (10):

10 $${a}_{ij}= \langle {{W}_{q}\otimes X}_{i },{{W}_{k}\otimes X}_{j }\rangle ,$$

where $\otimes$ denotes element-wise multiplication (Hadamard product) between vectors i.e. $${{W}_{q}\otimes X}_{i }=\left[\begin{array}{c}\begin{array}{c}{{x}_{i1}q}_{1}\\ {x}_{i2}{q}_{2}\\ .\end{array}\\ .\\ {{x}_{iq}q}_{d}\end{array}\right]$$ and $${{W}_{k}\otimes X}_{j }=\left[\begin{array}{c}\begin{array}{c}{{x}_{i1}k}_{1}\\ {x}_{i2}{k}_{2}\\ .\end{array}\\ .\\ {{x}_{iq}k}_{d}\end{array}\right]$$. The dot product or inner product is represented by $\langle .,.\rangle$.

The final attention will be modeled as Eq. (11):

11 $$\text{Attention}\left(\text{Q},\text{K},\text{V}\right)=\text{softmax}\left(\frac{{d}_{Q}A}{\sqrt{{d}_{Q}}}\right)\text{V}$$

To elaborate on the formulation, we can express the attention mechanism in terms of its components as Eq. (12):

12 $$Attention\left( {Q,K,V} \right) = \left[ {\begin{array}{*{20}c} {\begin{array}{*{20}c} {v_{1} soft\max \sqrt {d_{k} } W_{q} \otimes X_{1 } ,W_{k} \otimes X_{1 } } \\ {v_{1} soft\max \sqrt {d_{k} } W_{q} \otimes X_{2 } ,W_{k} \otimes X_{1 } } \\ \end{array} } & {\begin{array}{*{20}c} \ldots & {v_{n} soft\max \sqrt {d_{k} } W_{q} \otimes X_{1 } ,W_{k} \otimes X_{n } } \\ \ldots & {v_{n} soft\max \sqrt {d_{k} } W_{q} \otimes X_{2 } ,W_{k} \otimes X_{n } } \\ \end{array} } \\ {\begin{array}{*{20}c} \ldots \\ {v_{1} soft\max \sqrt {d_{k} } W_{q} \otimes X_{n } ,W_{k} \otimes X_{1 } } \\ \end{array} } & {\begin{array}{*{20}c} \ldots & { \ldots .} \\ \ldots & {v_{n} soft\max \sqrt {d_{k} } W_{q} \otimes X_{n } ,W_{k} \otimes X_{n } } \\ \end{array} } \\ \end{array} } \right]$$

Regarding the memory usage, the matrices memory can be approximated by their sizes, as illustrated in Eq. (13). Therefore, the total memory required for both matrices ${W}_{Q}$​ and ${W}_{K}$ is given by Eq. (14).

13 $${Memory}_{Q},{Memory}_{k}=n\times {d}_{Q} \left(For\,the\,Q\,and\,K\,matrices\right)$$

14 $${Memory}_{original}=2\times n\times {d}_{Q}$$

After compression, each compressed matrix ${{\acute{W}}_{Q}}$ and ${{\acute{W}}_{K}}$ consists of two components: a vector $q\in {R}^{{d}_{Q}}$ or $q\in {R}^{{d}_{Q}}$, which requires $d$ memory and a unit vector ${1}_{1\times {d}_{Q}}$, which is negligible in terms of memory. Thus, the memory required for both compressed matrices is given by Eq. (15), resulting in a compression ratio $R$ as defined in Eq. (16)

15 $${Memory}_{compressed}=2\times {d}_{Q}$$

16 $$R=\frac{{Memory}_{compressed}}{{Memory}_{original}}=\frac{2\times {d}_{Q} }{2\times n\times {d}_{Q}}=\frac{1}{n}$$

Regarding the computational requirements, the primary goal is to reduce the computational demand of self-attention layers, which rely on the matrix multiplication $Q{K}^{T}$ (refer to Eq. (1)). The complexity of Q and K is represented by $(n\times {d}_{Q})$, resulting in a final complexity of $O({n}^{2}\times {d}_{Q})$ for $Q{K}^{T}$. After compression, as per Eq. (4) and (5), both ${W}_{Q}$ and ${W}_{K}$ are compressed into matrices ${{\acute{W}}_{Q}}$ and ${{\acute{W}}_{K}}$, each represented by a vector of size ${d}_{Q}$ and a unit vector of size ${1}_{1\times {d}_{Q}}$. These compressed matrices effectively reduce the computation because instead of multiplying full-size matrices ${W}_{Q}\in {R}^{n\times {d}_{Q}}$ and ${W}_{K}\in {R}^{n\times {d}_{Q}}$, we use smaller vector-based operations. The compressed form involves vectors $q,k\in {R}^{d}$, and the unit vectors ${1}_{1\times {d}_{Q}}$, so the matrix multiplication of $\acute{Q}\acute{K}^{T}$ can be approximated as $O(n\times {d}_{Q})$.

#### Diagonal weight compression for V(DWC)

Building on the effectiveness of structured sparse matrix multiplication with diagonal storage schemes^35^, we were inspired to compress the $V$ matrix to reduce memory footprint and computational load without significantly impacting the model’s performance. This method demonstrated how focusing on key elements of the matrix, like diagonal components, can maintain essential information while eliminating redundancy. By leveraging this strategy, we aim to achieve similar efficiencies in the $V$ matrix, minimizing resource consumption without sacrificing accuracy. The compressed form of ${W}_{V}$ is updated in Eq. (17).

17 $${{\acute{W}}_{V}}={\text{diag}\left({\text{v}}_{1} ,{\text{v}}_{d} ,\dots ,{\text{v}}_{d}\right)}$$

where ${v}_{i} (for i=\text{1,2},\dots , d)$ represents the diagonal elements of the matrix.

The goal is to introduce sparsity in the matrix by converting it into a diagonal form, which retains essential information while eliminating redundancy in computations. This diagonalization simplifies the matrix operations, especially matrix multiplications, as most elements outside the diagonal are reduced to zero. This method ensures that the essential weights, which typically lie along the diagonal, are preserved while the less essential interactions between different dimensions (off-diagonal elements) are removed. By using a sparse representation of the matrix, we reduce the number of parameters and minimize the computational cost associated with matrix multiplications in the self-attention process.

Regarding memory usage, the original memory size of matrix $V$ is ${Memory}_{V}=n\times {d}_{V}$. After compression, only the diagonal elements need to be stored, so the memory usage for $V$ becomes ${Memory}_{V}={d}_{V}$. Thus, the total compressed memory usage can be represented by Eq. (18), resulting in a compression ratio $R$ as defined in Eq. (19)

18 $${Memory}_{compressed}=2\times {d}_{Q}+{d}_{V}$$

19 $$R=\frac{2\times {d}_{Q}+{d}_{V}}{2\times n\times {d}_{Q}+n{\times d}_{V}}$$

Regarding the computational requirements, to calculate the full attention computation (standard), we first detail the overall complexities of the operations. For the matrix multiplication $Q{K}^{T}$, the complexity is $O\left({n}^{2}\times {d}_{Q}\right)$. The $softmax$ operation is applied over each row of the resulting $Q{K}^{T}$ matrix, with complexity proportional to n for each row, resulting in $O\left({n}^{2}\right)$. Multiplying the attention scores with $V$ has a complexity of $O\left({n}^{2}\times {d}_{V}\right)$. Thus, the total computational complexity for standard self-attention is:$O\left({n}^{2}\times {d}_{Q}\right)+ O\left({n}^{2}\right)+O({n}^{2}\times {d}_{V})$. With compression applied to both the $Q$ and $K$ matrices using IRC, and to the $V$ matrix using Diagonal Weight Compression, the attention computation changes. The matrix multiplication $Q{K}^{T}$ is now $O\left(n\times {d}_{Q}\right)$ due to row replication. DWC reduces the computation for the multiplication with V to $O(n\times {d}_{V})$ because the multiplication involves only the diagonal elements rather than a full matrix. Therefore, the total computational complexity with compression is:$O\left(n\times {d}_{Q}\right)+ O\left({n}^{2}\right)+O\left(n\times {d}_{V}\right)$.

### Forward propagation pruning (FPP)

**Fig. 4:**
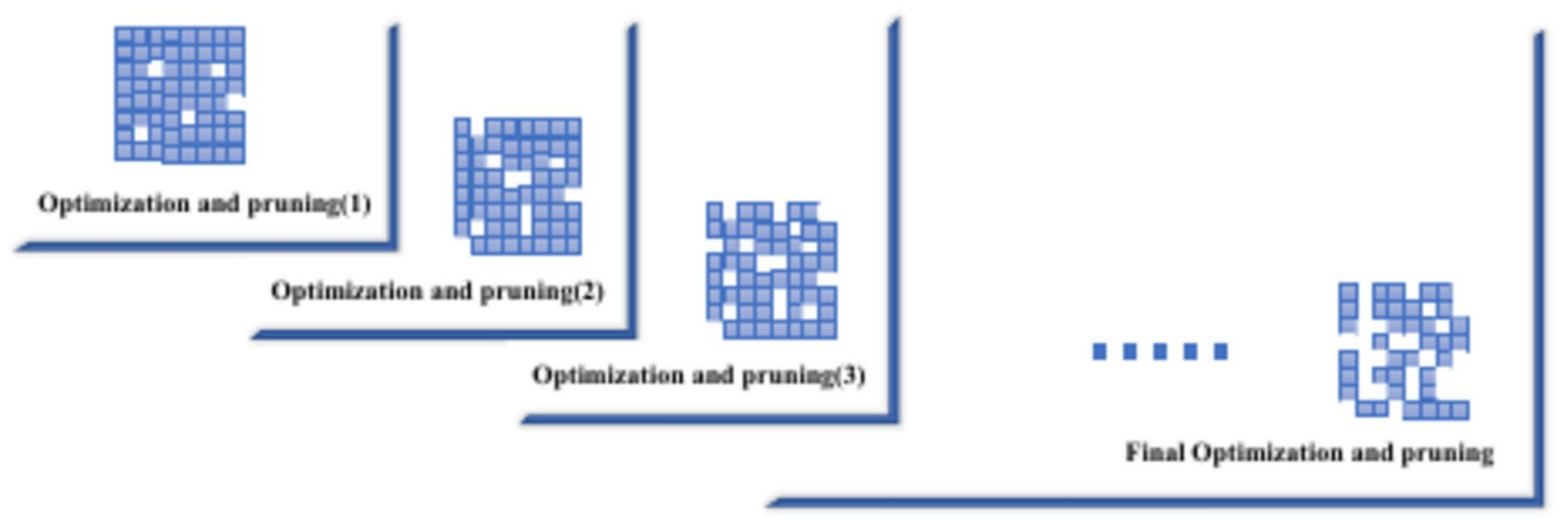
Forward Propagation Pruning process. In each forward step, the input weights are optimized and pruned based on the previous weights until we achieve the final optimization and pruning.

Our method, Forward Propagation Pruning, was inspired by the success of methods like Bonsai^36^, which demonstrated the power of efficient pruning techniques that rely solely on forward passes. Bonsai’s gradient-free, perturbative pruning showed that even without access to large-scale computational resources, it is possible to achieve impressive model compression without sacrificing performance. This inspired us to explore more layer-targeted pruning strategies, particularly for transformer architectures. While Bonsai focuses on perturbative pruning with forward passes, Forward Propagation Pruning takes this concept further by iteratively applying a pruning and optimization process specifically to the linear layers within transformers, as shown in Fig. 4.

Algorithm 2 represents the FFP process, which involves using the Adam optimizer to update layer weights across several iterations $T$ (maximum number of compression iterations). For each layer in the transformer ${Transf}_{layers}$, the weights ${layer}_{weights}$ are updated iteratively through ${Adam}_{iteration}$ steps. During each iteration $t$, positive and negative weights are optimized and sorted. A percentage ${r}_{pos}$ and ${r}_{nig}$ of the highest positive and lowest negative weights are pruned by setting them to zero and freezing them definitively, ensuring they are no longer updated. This process is repeated over multiple compression iterations. After pruning, the model is reinitialized and retrained using the updated compressed weights ${layer}_{weights}$. The model is then evaluated, and the compressed and optimized weights are integrated into the transformer layers. In the optimization process, we adopt a cumulative loss function *CumLossFunc*, to evaluate the model’s performance with a given set of parameters, similar to the approach used in^37^. This function considers the current model weights $\theta$, the training data batch $D$, and the previous loss value from the prior iteration ${L}_{i-1}$. It updates the batch loss by adding the current loss ${L}_{i}(\theta ,D)$ to the previous loss ${L}_{i-1}$ , allowing the Adam algorithm to consider the cumulative performance of the model across iterations during the optimization process. This strategy enhances the model’s learning by considering the overall trend in loss reduction rather than focusing solely on individual batches. We apply the Forward Propagation Pruning method to both the self-attention and feed-forward (FF) layers because these components are the most computationally expensive in transformer models. The self-attention layer calculates token relationships and has quadratic complexity $O({n}^{2})$ due to the interactions between all token pairs, making it resource intensive. Similarly, FF layers contain many of the model’s parameters $W$ and contribute significantly to memory and computation costs. Mathematically, the benefit of setting weights to zero can be illustrated by considering the number of non-zero parameters in the weight matrix $W$. If $W$ is a matrix of size $n\times m$, the total number of parameters is $n\times m$. After pruning and setting ${r}_{pos}$ of positive weights and ${r}_{neg}$ of negative weights to zero, the number of active (non-zero) weights ${r}_{0}$ is reduced to Eq. (20):

**Algorithm 2:**
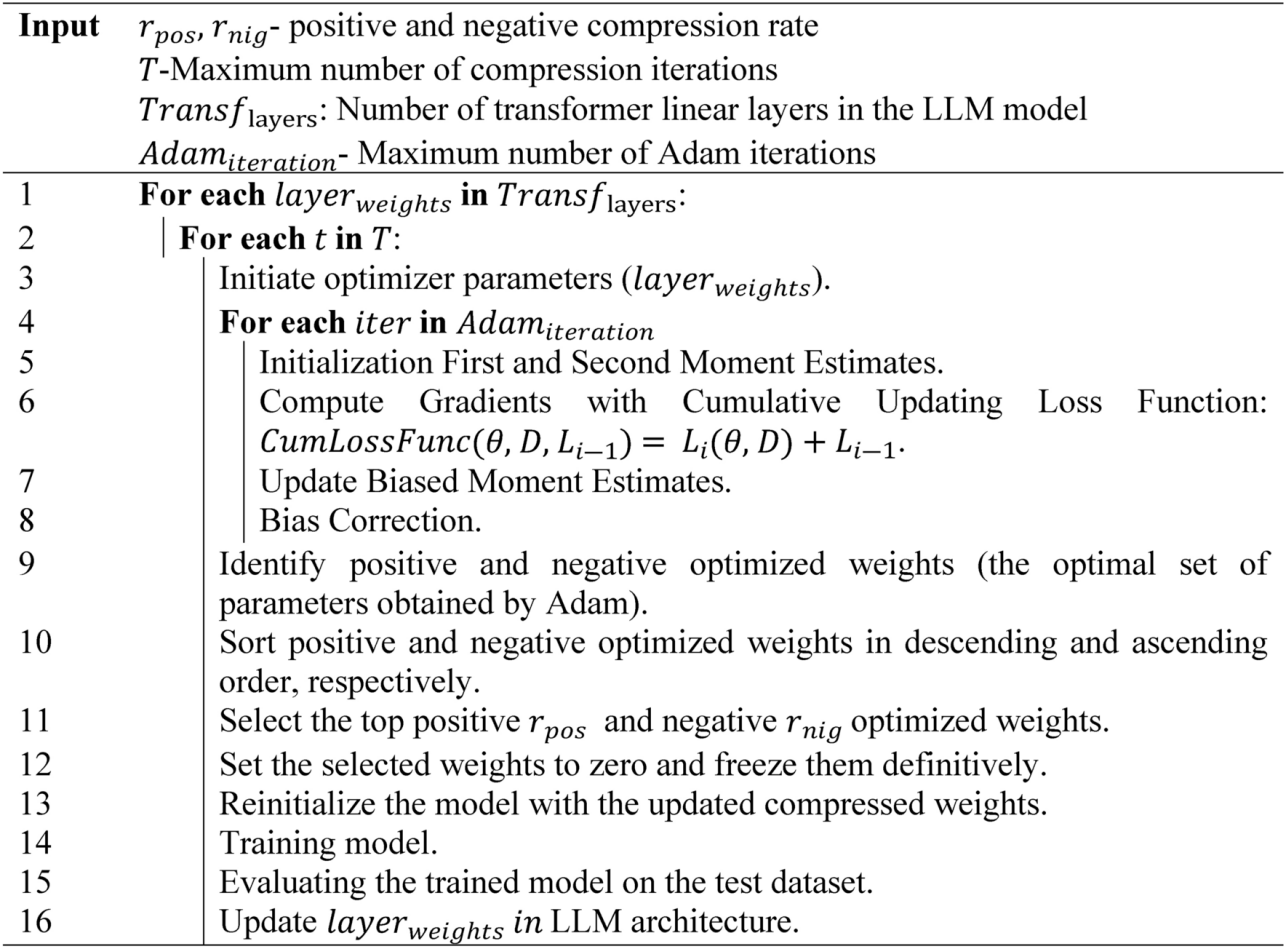
Forward propagation pruning algorithm.

20 $${r}_{0}=\left(1-\frac{{r}_{pos} }{100}\right)\times \left(1-\frac{{r}_{neg} }{100}\right)\times n\times m$$

This reduction decreases the number of multiplications required during inference, directly improving computational efficiency. Additionally, storing a sparse matrix (where many weights are zero) can take up significantly less memory, as only non-zero values need to be stored. This is often implemented with sparse matrix representations, which further optimize storage and computation.

## Training mechanism

In this section, we summarize the datasets we use. Then, we discuss the training process, covering hyperparameter settings and the setup for training. Lastly, we detail the metrics we use to evaluate model performance and effectiveness.

### Dataset

We evaluate our proposed approach on both Language Modeling (LM) and Text Classification (TC). For Language Modeling, we use the WikiText-2, WikiText-103^38^, and Penn Treebank (PTB) datasets^39^. For Text Classification, we utilize seven tasks from the General Language Understanding Evaluation (GLUE) benchmark^40^. These datasets are categorized into three types of tasks: single-sentence tasks, including linguistic acceptability (CoLA) and sentiment analysis (SST-2); similarity and paraphrasing tasks (MRPC and QQP); and inference tasks, including Natural Language Inference (MNLI and RTE) and Question Answering (QNLI). Additionally, we include the SMS Spam Collection dataset (SSC)^41^ to complement the GLUE benchmark, providing further insights into the algorithm’s performance across diverse text classification challenges. As shown in Table 1, the datasets vary in size, tasks, and application domains, which is critical for testing the effectiveness of our approach in various settings and domains. This diversity ensures a comprehensive evaluation of our approaches’ robustness and adaptability.

**Table 1:** Datasets descriptions and statistics. All tasks are binary classification, except MNLI (three classes).

Dataset	Corpus	Size	Task	Domain
Language modeling	WikiText-2	2 k	LM	Wikipedia
	WikiText-103	103 k	LM	Wikipedia
	Penn Treebank PTB	49 k	LM	English articles
Text classification	Corpus of Linguistic Acceptability (CoLA)	8.5 k	acceptability	Miscellaneous
	Recognizing Textual Entailment (RTE)	2.5 k	Natural Language Inference	Miscellaneous
	Microsoft Research Paraphrase Corpus (MRPC)	3.7 k	paraphrase	News
	Stanford Sentiment Treebank (SST-2)	67 k	sentiment	Movie reviews
	Multi-Genre Natural Language Inference (MNLI)	393 k	Natural Language Inference	Miscellaneous
	Question-answering Natural Language Inference (QNLI)	108 k	Question Answering (QA)	Wikipedia
	Quora Question Pairs (QQP)	364 k	paraphrase	Online QA
	SMS Spam Collection (SSC)	5.5 k	sentiment	Spam

### Training details

Regarding hardware, The CPU trials were conducted on a standard Windows (v8.1) server equipped with an Intel Core i7-9700 K processor (8 cores, 3.6 GHz base frequency, 12 MB cache), which provided robust performance for preprocessing and non-intensive computational tasks. GPU experiments were carried out using the Intel Data Center GPU Flex 140, featuring a state-of-the-art architecture optimized for deep learning workloads, with 16 GB of dedicated GDDR6 memory and support for hardware-accelerated AI inference. These platforms were selected to ensure a reliable and efficient environment for all experimental evaluations.

For the training details of our experimental results, we have devoted careful attention to specifying hyperparameters at every stage of developing and evaluating our proposal. These hyperparameters have been meticulously chosen to ensure optimal performance and generalization across various tasks. We demonstrate the effectiveness of our compression approach using GPT-2, a model comprising 12 layers, a hidden size of 768, and 12 attention heads, totaling approximately 124 million parameters. We selected GPT-2 for this study not only due to its widely recognized architecture and performance but also because it is an open-source model, providing us with flexibility and transparency. Regarding training hyperparameters, a typical learning rate is set around 5e-5, with batch sizes often allowing for 1,024 tokens during training. The model accommodates a maximum sequence length of 1,024 tokens, incorporating a dropout rate of 0.1 for regularization and a weight decay of 0.01 for the optimizer. Training generally spans 5 to 50 epochs, depending on the dataset size. The optimizer is Adam, configured with parameters β1 set to 0.9, β2 to 0.999, and ε to 1e-8. For baseline approaches, we adhere to the configurations outlined in the original papers. Table 2 presents the hyperparameters used to configure DistilGPT-2, KnGPT-2, QuantGPT, LightPAFF, and SparseGPT to validate the effectiveness of our approach.

**Table 2:** Evaluation hyperparameters.

Approach	Hyperparameters	Parameters setting
DistilGPT2	Batch Size	64
	Learning Rate	0.00025
	Learning Rate Schedule	α1: 0.5, α2: 0.5, α3: 0.5, and α4: 0.1
KnGPT2	Learning Rate	5e-5
	Batch Size	64
	Weight Decay	0.01
	Gradient Accumulation Steps	2
	Max Sequence Length	512 tokens
QuantGPT	Learning Rate	5e-5
	Batch Size	32
	Weight Decay	0.01
	Quantization Bits	16-bit
	Initialization Method	quantization-friendly
LightPAFF	Max Sequence Length	512 tokens
	Learning Rate	0.0005
	Weight Decay	0
	Temperature (τ)	0.1
	Momentum Coefficient (m)	0.5
	Optimizer	AdamW
	Batch Size	32
SparseGPT	Weight Decay	0.01
	Sparse Attention Heads	8
	Dropout Rate	0.3
	Gradient Accumulation Steps	4
	Learning Rate	5e-5
	Batch Size	32
Our Approach	Compression %	5% for positive and negative weights in each iteration
	Max iteration limit	*MaxIt* = 100
	Optimizer	Adam
	Learning Rate	5e-5
	Batch Size	32

### Evaluation metrics

We aim to provide a thorough assessment of our approach. We employ widely recognized metrics commonly used in the literature to accomplish this. To assess the prediction performance, we utilized the accuracy, precision, recall, and weighted F1-score. To evaluate the effectiveness of the optimization process, we conduct a dynamic loss test in which we utilize categorical cross-entropy, a suitable loss function for multiclass classification tasks.

For a more comprehensive evaluation, we introduce another metric known as perplexity. This metric is crucial for assessing language models such as the GPT, as it evaluates the model’s accuracy in predicting sequences of tokens^42^. Perplexity entails encoding the entire test dataset using the model’s tokenizer, segmenting it into numerous token segments, and calculating the average language modeling loss. The resulting exponentiated number represents the reported perplexity.

Additionally, we incorporate GPU speedup, latency, and throughput metrics, to evaluate further the overall performance and efficiency of the compression method. These metrics provide valuable insights into the impact of the compression method on the model’s speed and responsiveness across different hardware platforms. GPU latency refers to the time taken to process a batch of input on the GPU, while speedup is the ratio of the model’s performance improvement compared to Simple GPT-2. Fine-tuning throughput is measured in sequences per second during the fine-tuning process^43^, with higher throughput typically indicating faster training and fine-tuning, particularly in lighter or pruned models.

## Results and discussion

To demonstrate the effectiveness of our compression approach, we begin by analyzing how the compression methods affect model weights and their subsequent impact on loss, accuracy, and overall model performance.

**Fig. 5:**
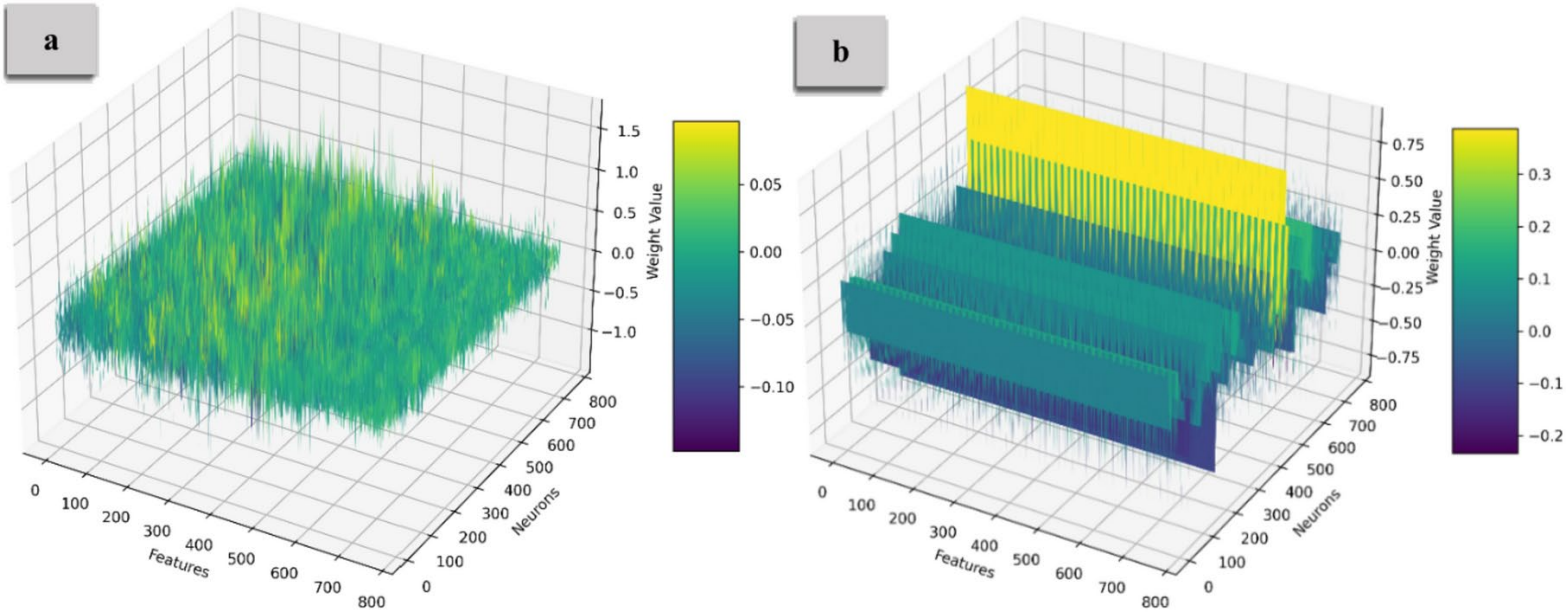
The weight distribution of the Query matrix. Before compression, the weights exhibit high variability, creating a complex 3D landscape. After applying Identical Row Compression, many rows become identical, resulting in a flatter and more uniform distribution along specific dimensions.

**Fig. 6:**
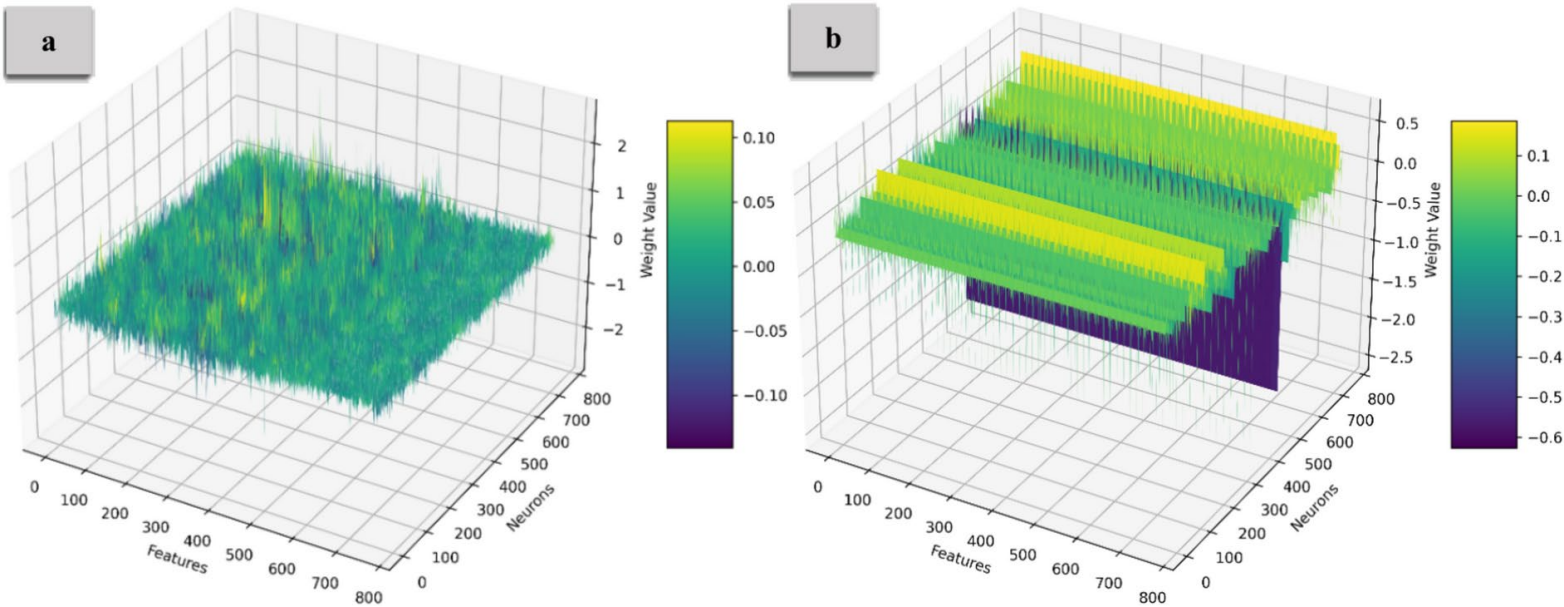
The weight distribution of the Key matrix. After applying Identical Row Compression, many rows become the weight distribution flattens, creating a more uniform arrangement.

**Fig. 7:**
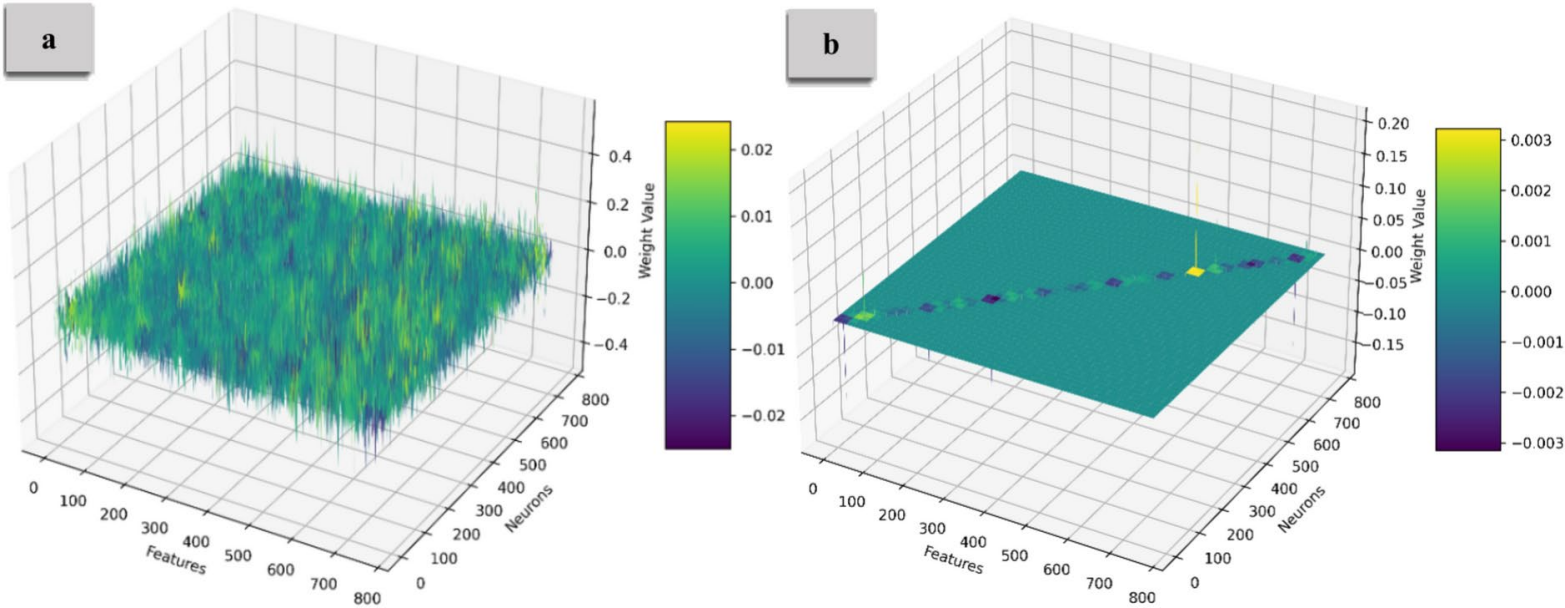
Weight distribution of the Value matrix. The original dense matrix appears as a fully populated 3D cube. At the same time, the compressed version transforms into a diagonal structure, retaining only the diagonal elements and significantly reducing parameters.

Figure 5 and 6 illustrate the weight distribution of the Query and Key matrices, with Part (a) showing the distribution before compression and Part (b) displaying it after compression. Before compression, the weights exhibit high variability across multiple dimensions, creating a complex 3D landscape. After applying Identical Row Compression, many rows become identical, flattening the distribution along certain dimensions and transforming the weight landscape into more uniform, parallel planes. Figure 7 illustrates the weight distribution of the Value matrix. The original dense matrix is represented as a fully populated 3D cube, with each cell containing weight values reflecting the attention across input tokens. After Diagonal Weight Compression, the matrix transforms into a diagonal structure, visualized as a cube where only the diagonal elements (from the top-left to the bottom-right) retain non-zero values, while the rest are zero. This transformation highlights the preserved weights, significantly reducing parameters and computational demands, with sparsity introduced through compression.

**Fig. 8:**
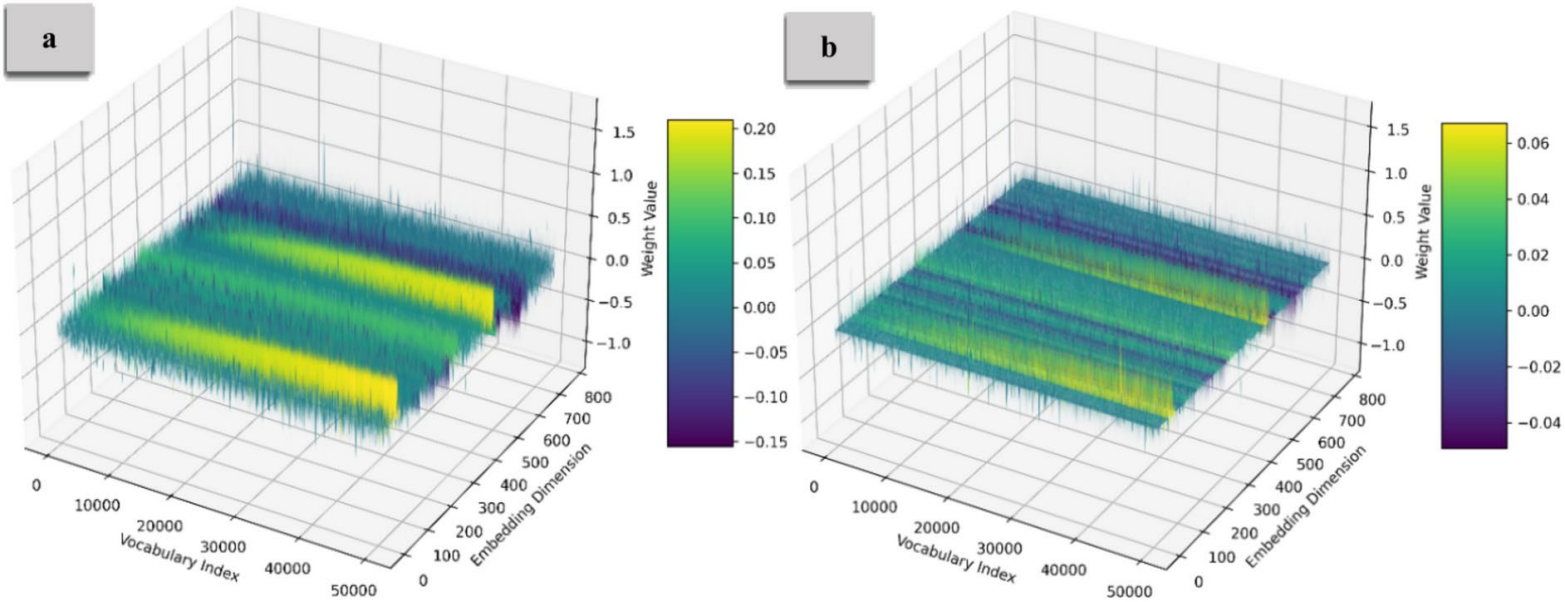
The embedding layer’s weight distribution. Part (**a**) displays a dense distribution before compression, while Part (**b**) shows a sparse distribution after compressing 70% of the weights. This highlights that the most influential weights are maintained in a sparse configuration.

**Fig. 9:**
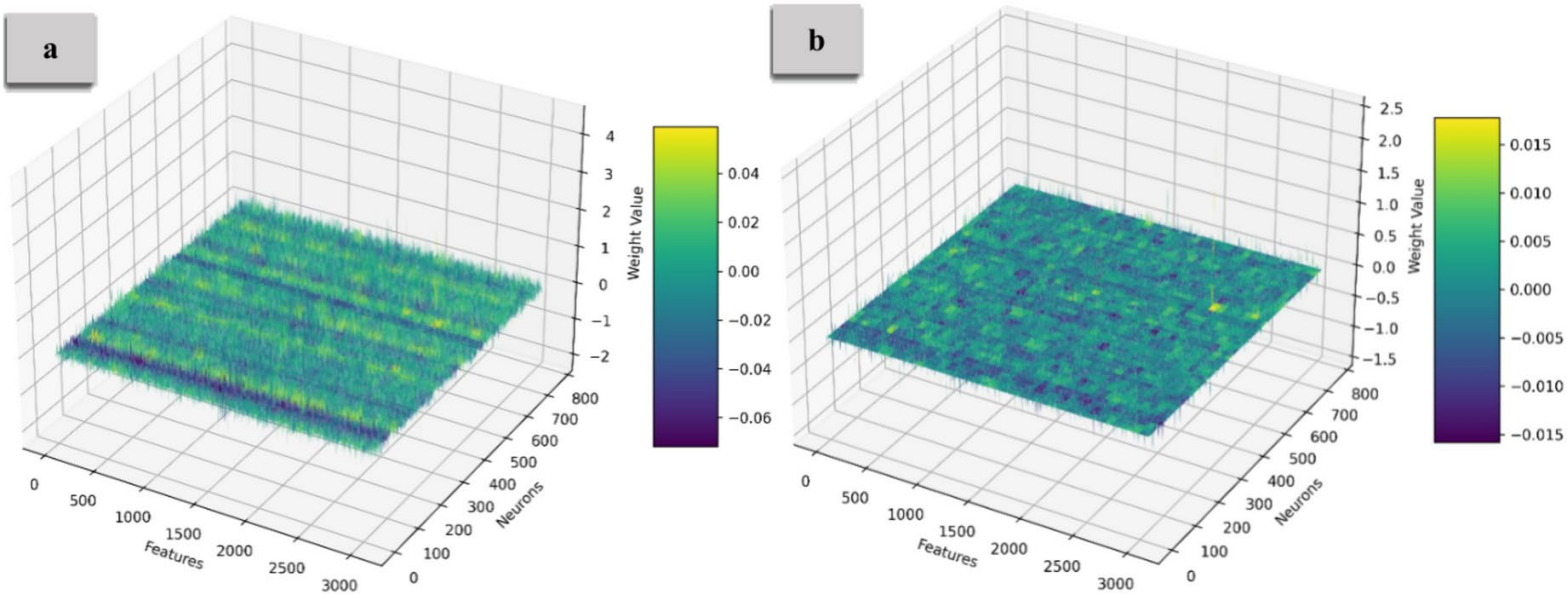
The FF layer’s weight distribution. The shift to sparsity emphasizes key weights, simplifying the model architecture and improving inference speed and generalization capabilities.

Figure 8 and 9 illustrate the weight distribution of the Embedding Layer and FeedForward weights, with Part (a) showing the distribution before compression and Part (b) displaying it after compression. For the Embedding Layer, clusters of weights highlight specific features or tokens, while the FeedForward Layer displays more significant variability, reflecting complex feature interactions. The density of points suggests that many weights contribute to decision-making. After applying the compression that zeroes out 70% of the weights, the 3D representation reveals a significant shift to sparse distributions. Many points are removed, leaving concentrated regions in the 3D space where the remaining weights retain substantial influence. This sparsity simplifies the model’s architecture, leading to faster inference time. The transition from dense to sparse weight distributions suggests that many weights may be redundant. Relying on the most critical weights enhances the model’s generalization capabilities, indicating areas of strong feature interactions or dependencies.

**Fig. 10:**
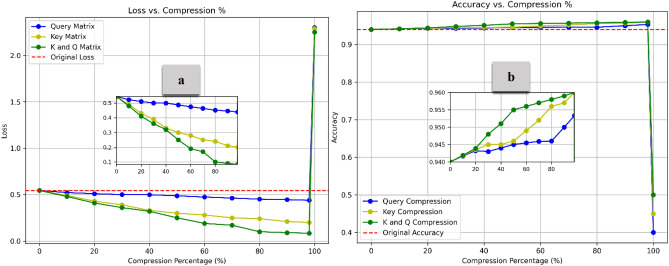
Loss and Accuracy vs. Compression % under Identical Row Compression. All identical row compression methods improve performance, with the combined K and Q Compression achieving the best 99% compression. Key compression outperforms Query Compression, emphasizing the Q matrix’s sensitivity to compression.

To clarify the impact of the compression methods on loss and overall model performance, we conducted a detailed analysis of each method employed in the overall compression process. Figure 10 presents the Loss (Part a) and Accuracy (Part b) distributions across different compression rates for the Query and Key matrix compressions. Compared to the baseline, all identical row compression methods achieve high compression rates without sacrificing performance. Notably, performance increases across all methods as compression intensifies, with the combined compression of the K and Q matrices showing the most substantial improvement, reaching a peak accuracy of approximately 0.96 at 99% compression. Key outperforms Q compression in accuracy gains, further emphasizing Q compression’s minimal improvement across both metrics. The Q matrix tends to be more sensitive to compression than the K matrix because queries directly impact what part of the input the model focuses on. Compressing the K matrix (especially using methods like identical Row Compression) can often tolerate more compression without as significant a loss in performance because key relationships between tokens tend to be more redundant.

**Fig. 11:**
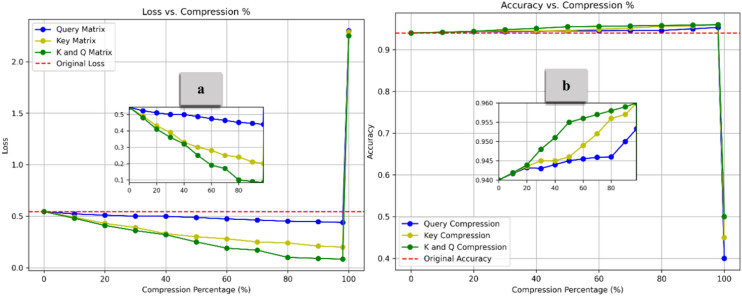
Loss and Accuracy vs. Compression % under Diagonal Weight Compression. The combined approach achieves the highest accuracy, highlighting the effectiveness of compressing both the upper and lower parts of the V matrix simultaneously.

Figure 11 compares the Loss (Part a) and Accuracy (Part b) for the Value matrix under Lower, Upper, and combined Triangular Compressions. Compared to the baseline, all diagonal weight compression methods achieve high compression rates without compromising performance. Accuracy improves overall as compression increases, with Upper and Lower Triangular Compression achieving the highest accuracy gains, approximately 0.975 at 98% compression. Upper Triangular outperforms Lower Triangular compression in accuracy gains, further emphasizing Lower compression’s minimal improvement across both metrics. Compressing the lower part of the V matrix in self-attention is sensitive because it affects how information from previous tokens is passed forward^44^, potentially disrupting dependencies. Adopting both upper and lower triangular compression preserves more structural information, ensuring better representation and flow between tokens. This balanced approach enhances performance while maintaining high compression rates, making it more effective than compressing one part alone.

**Fig. 12:**
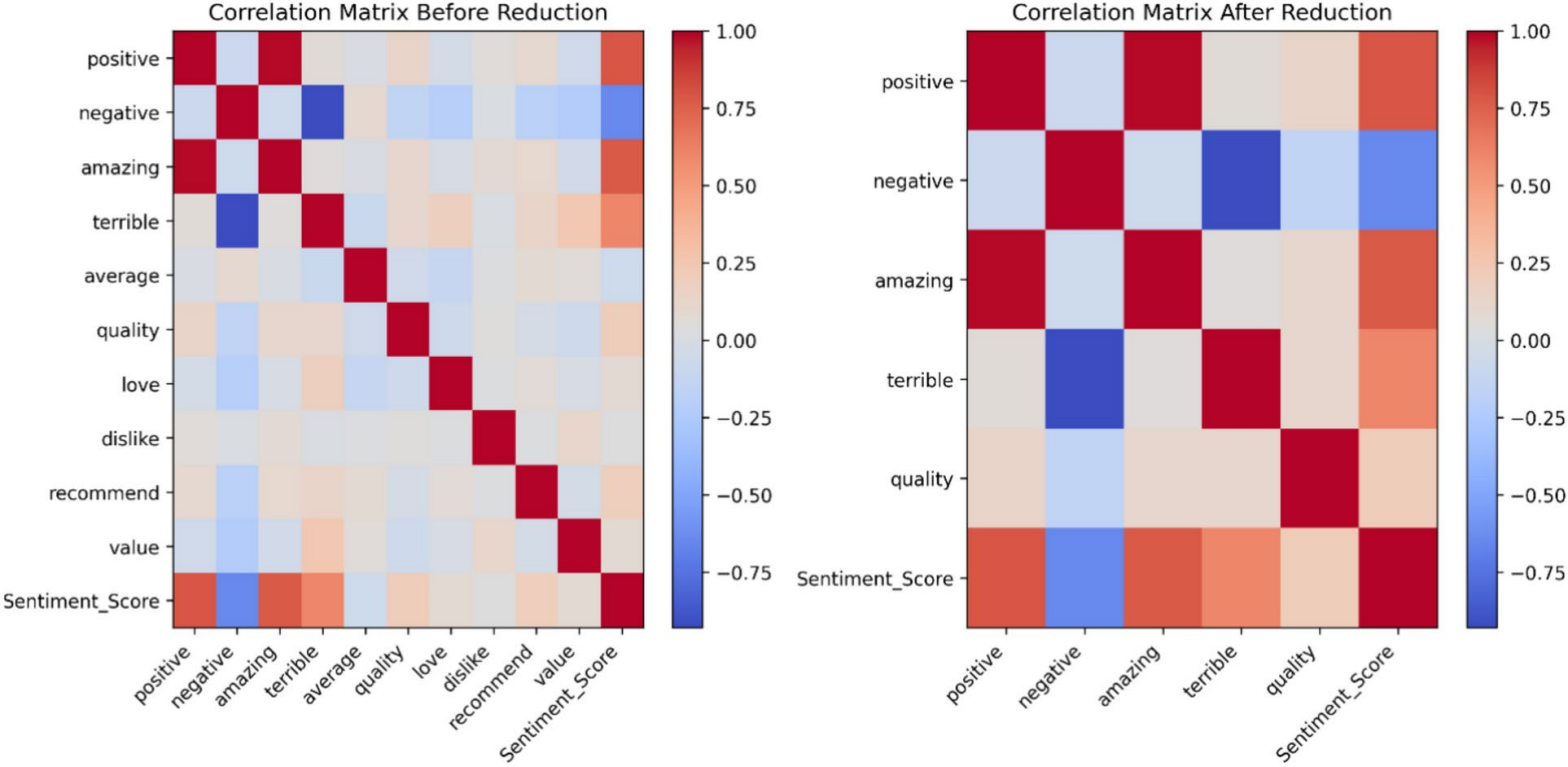
Example of Correlation Matrix Behavior (Sentiment Analysis Case). The correlation matrix before feature reduction includes all features, highlighting spurious correlations between some (e.g., “positive” and “amazing”). After feature reduction, only the most relevant features are retained, minimizing irrelevant correlations and enhancing model interpretability.

To better understand how our mathematical model improvements help maintain minimal loss (Fig. 10 and 11), we examine the correlation of our model before and after pruning, as shown in Fig. 12. The reduction method appears to have compressed the correlation structure, removing extreme values and reducing redundancy between the sentiment-related attributes^45^. The minimal correlation loss despite substantial compression from IRC and DWC can be attributed to how both compression methods focus on retaining the most crucial components of the self-attention mechanism while simplifying the model’s computations. In IRC, the Q and K matrices are compressed by making all rows identical for each token. This drastically reduces computational complexity, but crucially, each token’s attention relationship with others is still preserved through the scalar weight assigned to it^46^. By reducing the variability in the Q and K matrices, the compression technique captures the core relationships between tokens, while eliminating redundancies. This ensures that the model can still compute meaningful attention scores without the need for full matrix representations. In DWC, the compression method converts the V matrix into a diagonal form, which focuses on retaining only the most essential diagonal components. Diagonal elements typically capture the strongest relationships, and the off-diagonal elements, which usually represent less significant pairwise interactions, are discarded. This effectively reduces the number of parameters and the complexity of matrix multiplications without sacrificing the primary interactions that influence attention. Both methods focus on structured sparsity and controlled redundancy, which ensure that the most informative aspects of the Q, K, and V matrices are retained while unnecessary computations are eliminated. The result is a model that can process data with much lower memory and computational overhead, while still preserving the vital attention relationships between tokens. The preservation of these key relationships—particularly those between relevant token pairs—explains why the overall accuracy loss is minimal, even with the aggressive compression. In other words, while much of the redundant data is removed, the parts that are essential for effective self-attention computation remain intact, thereby allowing the model to maintain its performance.

**Fig. 13:**
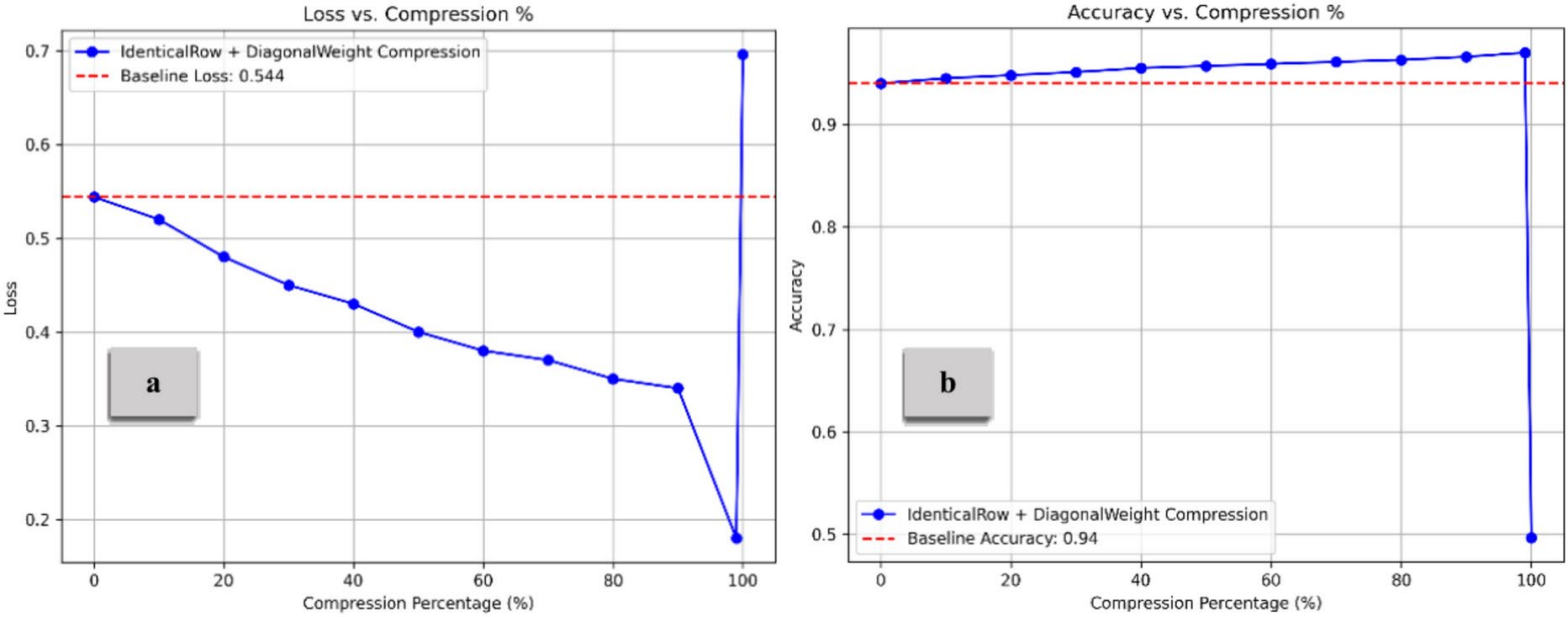
Loss and Accuracy vs. Compression % under IRC and DWC combination. The model’s performance improves with compression until it reaches a high compression rate, which subsequently decreases its effectiveness.

**Fig. 14:**
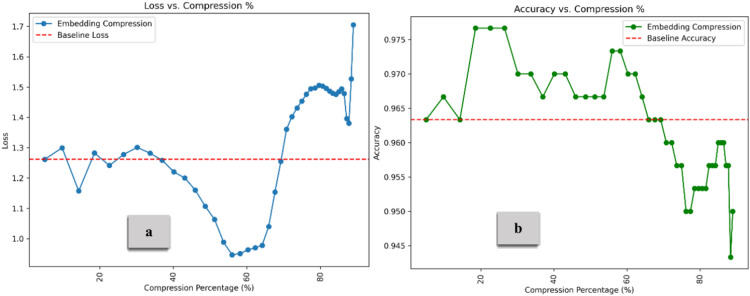
Loss and Accuracy vs. Compression % under Forward Propagation Pruning for the Embedding layer. The results indicate that embedding compression achieves lower loss and higher accuracy than the baseline, up to 70% compression, before a sharp decline at higher compression rates.

**Fig. 15:**
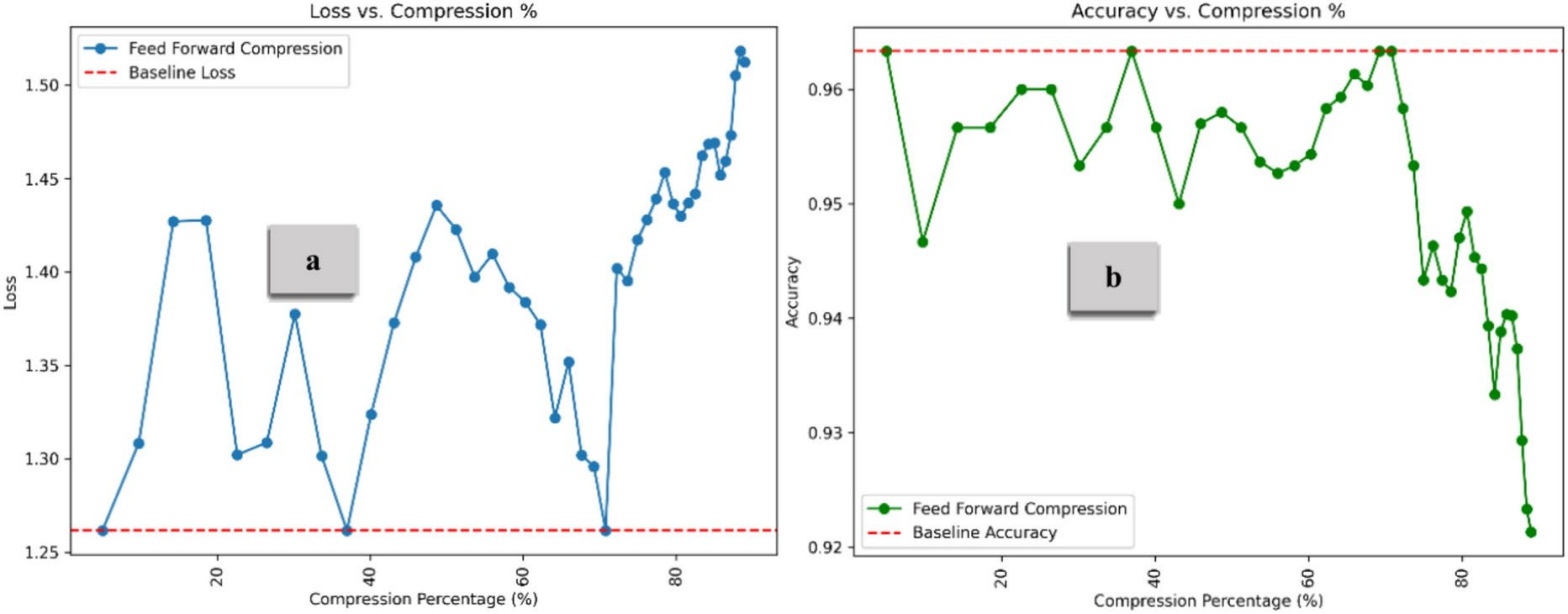
Loss and Accuracy vs. Compression % under Forward Propagation Pruning for the FeedForward Layer. This figure illustrates a non-linear relationship, with loss increasing at higher compression levels. Accuracy stays above the baseline with moderate compression but drops significantly at extreme levels.

Figure 13 illustrates the combination impact of Diagonal Weight Compression (for the V matrix) and Identical Row Compression (for the Q and K matrices). As the compression percentage increases, accuracy improves, peaking at approximately 0.97 before sharply declining at nearly 99% compression. The DWC simplifies the V matrix by reducing its variability, which can lead to more efficient information retention in the early stages of compression. This maintains the model’s capacity to focus on key elements without unnecessary complexity. Similarly, IRC reduces noise by replicating a single row across all Q and K matrix rows. This helps to preserve key aspects of the attention mechanism while reducing the model’s sensitivity to noise or irrelevant data. The accuracy increases at moderate compression levels as extraneous information is removed without impacting performance. As compression reaches extreme levels, too much information is discarded. Zeroing rows too aggressively reduces the diversity in attention, making it difficult for the model to distinguish between different elements in the input. The model loses the ability to capture rich dependencies in the data fully^47^. The self-attention mechanism becomes overly simplistic, leading to a significant loss in performance at very high compression. As illustrated in Fig. 14, The embedding compression method demonstrates lower loss than the baseline up to approximately 70% compression, with the lowest loss occurring around 55–57%. After 70%, the loss increases sharply, reaching a peak of about 1.7. The compression method consistently performs above the baseline (set at 0.963) for most of the compression range. Interestingly, with a moderate compression rate of 5% to 65%, model performance remained stable and improved, highlighting the potential for optimized efficiency. However, beyond 70% compression, accuracy declines rapidly, dropping to around 0.945 at the highest compression levels. On the other hand, Fig. 15 demonstrates the results for feed-forward compression, with loss exhibiting changes but trending upward as compression increases, especially beyond 70%. Regarding accuracy, the compression method maintains performance above the baseline by up to 70% compression. However, accuracy declines sharply beyond this threshold, dropping to around 0.92 at the highest compression levels. This pattern suggests that moderate compression improves model efficiency and performance, but extreme compression reduces the model’s ability to retain critical information, leading to degraded accuracy and increased loss.

**Fig. 16:**
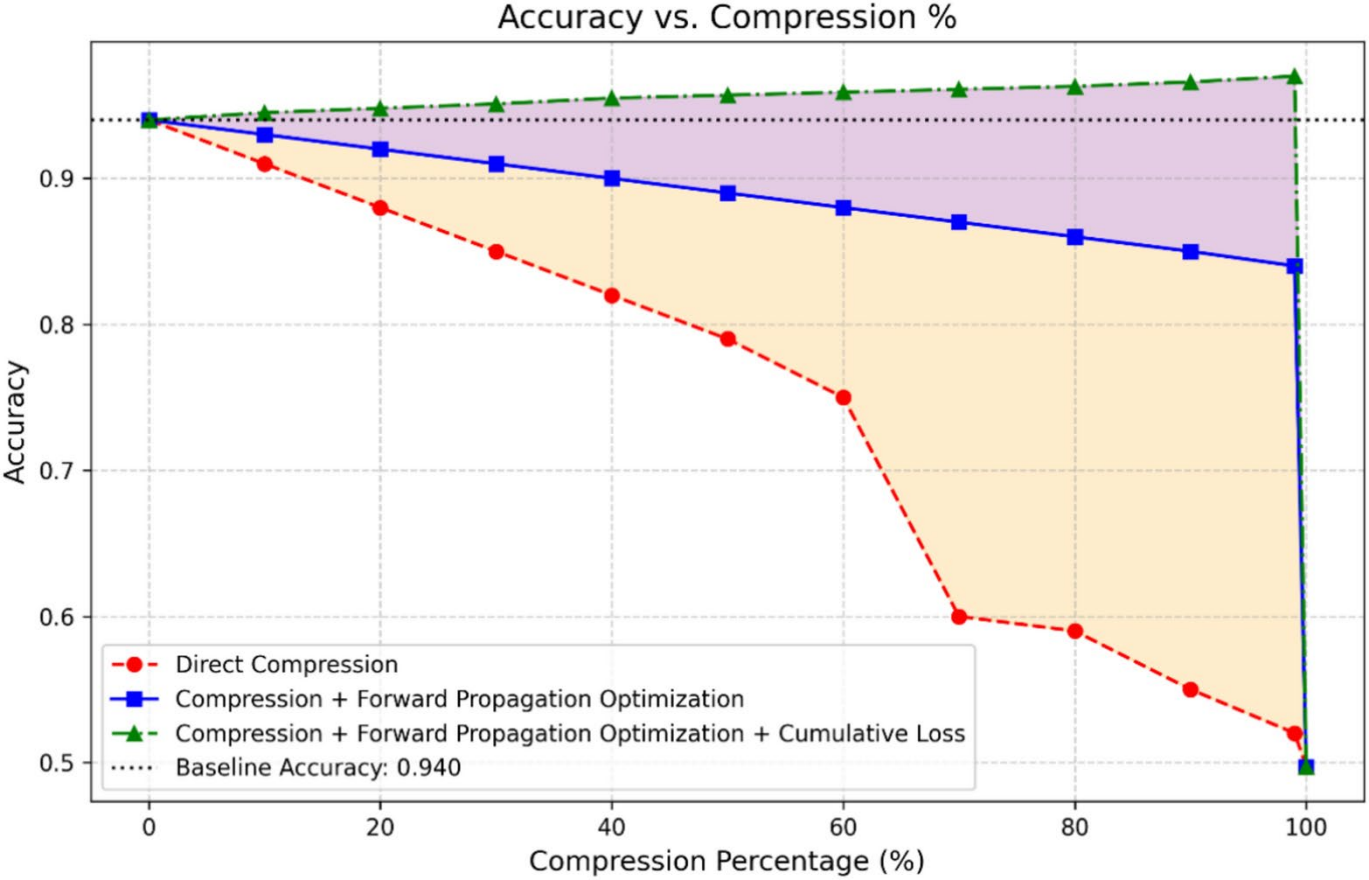
The relationship between the compression percentage and the accuracy of the model under the IRC and DWC combination, comparing direct compression, compression with forward propagation optimization, and compression with forward propagation optimization combined with the cumulative loss function.

From Figs. 13, 14, and 15, it is observed that the accuracy increases with a higher compression ratio. To better understand the reasons behind this behavior, we conducted a detailed analysis of the various sub-approaches used in our method. The recursive compression algorithm dynamically adjusts the compression rate based on performance evaluation after each step. This incremental optimization process fine-tunes the model weights throughout the compression, allowing the algorithm to find a more effective representation of the model by balancing compression and accuracy. As the algorithm progresses, it converges towards an optimal compressed state, where the model’s performance improves. In each iteration, the model reoptimizes its connections using sub-optimizer steps of Adam, applying a cumulative loss function to reduce errors and ultimately achieve the optimal set of parameters. Figure 16 demonstrates the effects of different compression techniques on model accuracy. Direct compression shows a steady decline in accuracy as the compression percentage increases. Compression with forward propagation optimization maintains accuracy much better as compression increases. Compression with forward propagation optimization and a cumulative loss function actually improves accuracy as compression increases, reaching nearly the baseline accuracy of 0.940 even at 90% compression. The key point is that the recursive compression algorithm, which adjusts the compression rate based on performance evaluation after each step, is able to find an optimal compressed state that balances compression and accuracy. The cumulative loss function further improves this process, allowing the model to converge towards an optimal set of parameters that maximizes accuracy even under high compression rates. This demonstrates the effectiveness of the methodology in maintaining and even improving model performance through the compression process, rather than simply seeing a linear decrease in accuracy as the compression percentage increases.

To further evaluate the model’s effectiveness, we conducted an ablation study to assess the impact of each compression method individually. This allowed us to understand the overall benefit of each method when applied to different layers, such as the embedding layer, feed-forward layers, and self-attention layers (including Q, K, and V weights). Tables 3 and 4 provide a detailed comparison of various model compression methods, focusing on their impact on model performance and size. Most compression methods preserve or slightly improve these metrics. The overall compression demonstrates promising results, achieving compression rates of up to 70.69% while maintaining performance. Block-level compression ranges from 70 to 99%, while model-level compression achieves 11% for IRC, 6% for DWC, 22% for ${FFP}_{emb}$, and 32% for ${FFP}_{FF}$. Notably, these methods also boost accuracy by 1% to 3%, highlighting their effectiveness. The trade-off between compression rate and model performance demonstrates the potential for deploying models on resource-constrained devices or reducing computational costs in large-scale applications without compromising accuracy.

**Table 3:** Comparative analysis of the effect of the proposed compression methods, where $C\%$ represents compression rate and ${FFP}_{FF}$ and ${FFP}_{FF}$ represents the Forward Propagation Pruning method applied to the embedding and feedforward layers. Compared to the base model, the compressed model (70%) achieves a significantly better compression rate, maintaining almost accuracy, F1 score, recall, and precision.

Method		${\varvec{C}}\%$	Accuracy	Loss	F1 score	Recall	Precision
Original model		0%	94.67	0.54	0.945	0.947	0.944
WMF	IRC	~ 11%	96.01	0.19	0.958	0.950	0.956
	DWC	~ 6%	**97.50**	**0.08**	**0.969**	0.964	0.960
FFP	FFP_emb_	~ 22%	96.43	1.25	0.958	0.959	0.957
	FFP_FF_	~ 32%	96.66	1.30	0.965	**0.966**	**0.964**
Overall Compression		~ 70%	94.25	0,60	0.940	0.941	0.939

**Table 4:** Compression rate of GPT, where **C%UB** represents the Compression Percentage Under Block and **T%** represents the Total Percentage.

Model		Number of Parameters		C%UB		T%
		Before	After			
Original model		124,235,010		0%		
WMF	IRC	14,155,776	18,432	99.98%		11.38%
	DWC	7,077,888	9,216	99.86%		5.68%
FPP	FPP_emb_	38,602,560	11,580,768	70%		21.75%
	FPP_FF_	56,669,184	17,000,748	70%		31.9%
Overall Compression						70.69%

To understand the overall benefit of each method when applied to different layers, such as the embedding layer, feed-forward layers, and self-attention layers (including Q, K, and V weights), we evaluate the performance of our compressed model on two domains, starting with language modeling datasets as presented in Table 5. The models are initialized, trained on three datasets, and evaluated using the provided test set. Lower perplexity scores indicate better performance, reflecting the model’s confidence and accuracy in predicting the next token in a sequence. Our approach consistently achieves the lowest perplexity scores across all datasets (14.6, 12.7, and 14.7, respectively), demonstrating superior performance in language modeling tasks. In contrast, SparseGPT exhibits higher perplexity scores, suggesting more incredible difficulty predicting the next token^36^. Additionally, some models, such as KnGPT2 and LightPAFF, need more data for the PTB dataset^32^, making a comprehensive comparison challenging. Next, the performance of the approaches is evaluated on test sets of seven datasets of the GLUE benchmark and SSC dataset (Table 6). Our approach shows strong performance across most datasets, achieving the highest scores in 4 out of 8 datasets and the highest average score (84.31% and 85.40% for 70% and 30% compression rates, respectively). KnGPT2 and LightPAFF demonstrate competitive performance, often scoring close to the top in several datasets. SparseGPT or QuantGPT show variable performance, excelling in some datasets (e.g., SST-2 or QNLI) but lagging in others. Our model demonstrates superior performance in language modeling (lowest perplexity) and text classification tasks (highest average accuracy). This suggests that an effective balance between model complexity and task performance has been found. Some models show strengths in specific datasets or tasks. For example, SparseGPT performs well in language modeling but has more variable results in classification tasks^4^. This highlights the performance of our compression approach across both language modeling and classification tasks, suggesting it has good generalization capabilities^11^, making it potentially valuable for a wide range of NLP applications.

**Table 5:** Perplexity comparison across various approaches on language modeling datasets (WikiText2, WikiText103, and PTB). Our approach achieves the lowest perplexity across all datasets, significantly outperforming baseline models such as GPT-2, DistilGPT2, and SparseGPT.

Approach	Language Modeling Dataset		
	WikiText2	WikiText103	PTB
GPT-2	35.57	20.2	37.2
DistilGPT2	-	21.1	-
KnGPT2	21.94	20.5	-
QuantGPT	17.30	16.12	16.98
LightPAFF	18.8	16.4	22.8
SparseGPT	37.5	22.3	40.6
Our approach	14.6	12.7	14.7

**Table 6:** Model performance on text classification datasets across various compression methods. For SST-2, SparseGPT reports the best accuracy. For QNLI, QuantGPT achieves the highest accuracy. For all other tasks, our proposed approach (with compression of 70%) demonstrates the highest accuracy, with only a 2.5% decrease while outperforming the baselines.

Approach	Text Classification Dataset								
	CoLA	RTE	MRP	SST-2	MNLI	QNLI	QQP	SMS	Avg
Base model	69.0	75.9	89.5	94.8	85.7	92.7	92.5	94.6	86.86
DistilGPT2	32.4	61.9	84.3	90.8	79.5	85.4	87.3	89.6	76.3 ($\downarrow 10.5$)
KnGPT2	36.7	64.4	84.5	89.0	78.4	85.6	86.1	88.5	76.6 ($\downarrow$ 10.2)
QuantGPT	56.7	71.5	87.2	92.0	84.1	**91.0**	89.7	89.8	82.7 ($\downarrow$ 4.2)
LightPAFF	58.2	73.5	86.5	89.5	76.5	90.2	88.6	91.3	81.7 ($\downarrow$ 5.0)
SparseGPT	57.7	62.3	86.0	**92.2**	74.3	89.2	88.5	92.7	80.36 ($\downarrow$ 6.5)
Our approach (30%)	**65.5**	**74.3**	**88.8**	92.1	**85.1**	90.7	**92.3**	**94.3**	**85.40(**$\downarrow$** 1.4)**
Our approach (70%)	60.9	73.9	88.5	91.4	80.8	89.2	90.5	94.2	84.31 ($\downarrow$ 2.5)

We provide examples of sentences generated by our model compressed using the prompts « Once upon a time » and «The Industrial Revolution» in Table 7. We tried to ensure minimal overlap between these generated sentences and the information in the tuning corpus, demonstrating that the information originates from the original model rather than the tuning corpus. Regarding linguistic flow and topical consistency, the sentences generated by the pruned model are comparable to those from the original model. The results indicate that our compression method effectively maintains coherence and fluency, showcasing a clear narrative flow in short and long outputs despite the high compression level.

**Table 7:** Generation Examples from the Baseline Model(GPT) vs. Our 70%-Compressed Model. The underlined text represents the prompt for text generation. The results show that our compression method maintains coherence and fluency, especially in short outputs despite the high level of compression.

Model		Generated Short Text	Generated Long Text
Original Model		Once upon a time, a little girl found a hidden treasure in the forest	The Industrial Revolution, which began in the late eighteenth century, transformed economies from agrarian to industrial. It led to mass production, urbanization, and significant technological advancements
70%-compressed Model	Prompt 1	Once upon a time, in a land far away, there lived a brave knight who sought exciting adventures	The Industrial Revolution marked a turning point in history. With new inventions, such as the spinning jenny and the steam engine, production increased, leading to urban growth
	Prompt 2	Once upon a time, there was a curious kitten who wanted to explore the wonders of the world	The Industrial Revolution had a profound impact on society. It shifted labor from farms to factories, significantly altering lifestyles
	Prompt 3	Once upon a time, a little girl discovered a hidden gem in the woods	The Industrial Revolution began in the late 1700s and involved major technological advances. While it led to great progress and wealth for some, it also resulted in pollution and difficult labor conditions for many workers
	Prompt 4	Once upon a time, there was a wise old owl who knew the secrets of the forest	The Industrial Revolution changed many aspects of life. Factories replaced small workshops, leading to increased productivity
	Prompt 5	Once upon a time, in a small village, children played joyfully under the sun	The Industrial Revolution, which began in the late 1700s, was an important time. It changed how things were made

**Fig. 17:**
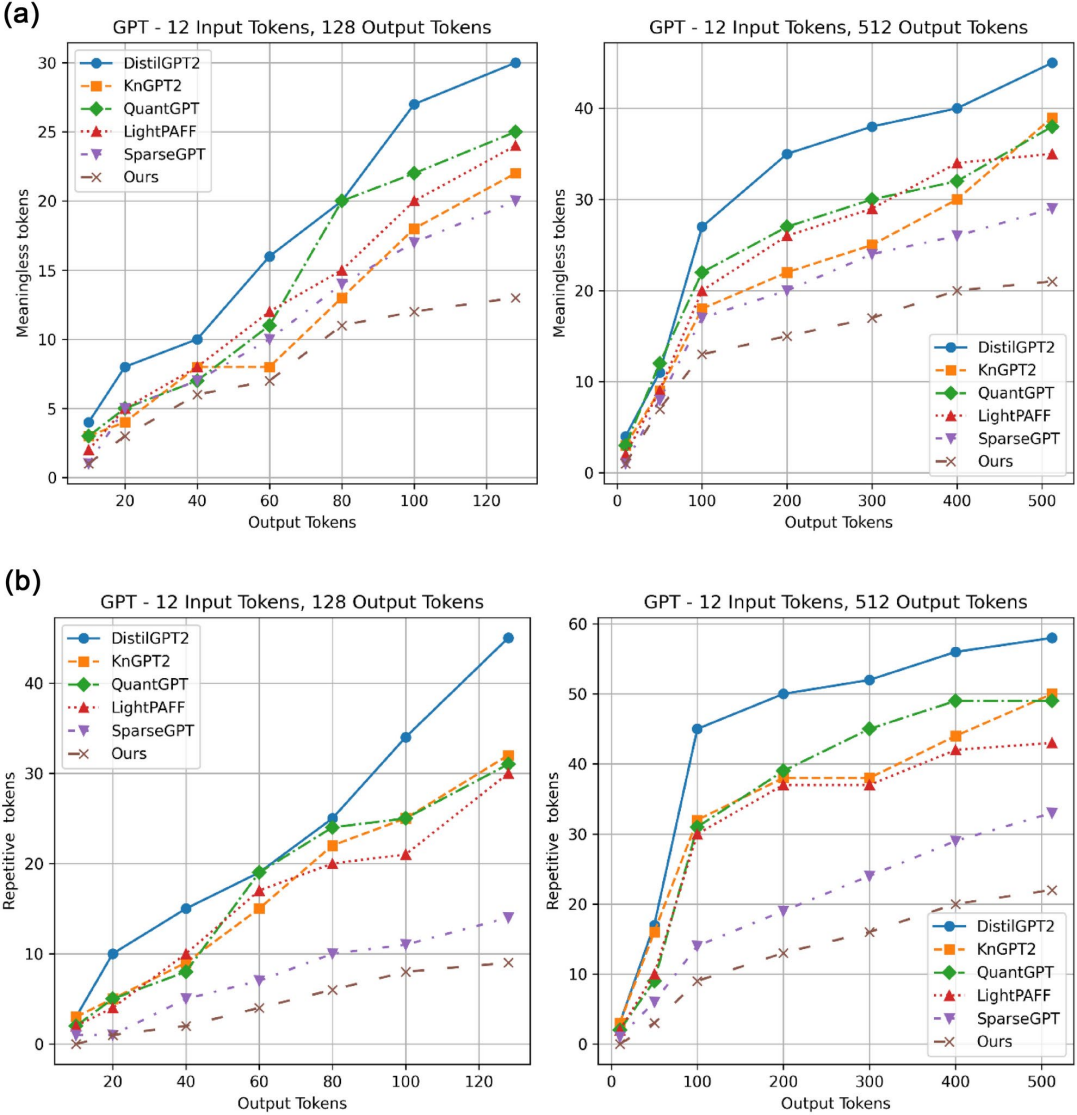
(**a**). Meaningless tokens of our 70% compressed model vs. baseline compression methods such as DistilGPT2, KnGPT2, QuantGPT, LightPAFF, and SparseGPT. For 128 and 512 output tokens, the 70% compressed model gives the least meaningless, outstanding baseline models in short and long sentences. (**b**). Repetitive tokens of our 70% compressed model vs. baseline compression methods. For 128 and 512 output tokens, the 70% compressed model gives the least repetitive tokens, outstanding the baseline models in short and long sentences. The result reports that the performance of pruned models deviates from ours, particularly when generating lengthy sentences, which create more meaningless or repetitive tokens.

Table 8 compares various compression approaches applied to GPT-2, highlighting their effects on performance metrics. The new approach (70% compression) outperforms others in speedup and fine-tuning throughput. While DistilGPT2 achieves moderate compression with good speedup and throughput, QuantGPT prioritizes compression at the expense of some performance. Overall, the new compression approach significantly enhances efficiency and performance, enabling faster and more resource-efficient deployment of large language models. Figure 17a and b compare the performance of various GPT compression methods regarding meaningless and repetitive tokens across different input and output token lengths. The right section focuses on short outputs(128 output tokens), allowing us to evaluate how the model handles small, simple queries and whether the compression method preserves performance. In contrast, the left section represents longer sequences(512 output tokens), often encountered in more complex tasks such as text summarization, document generation, or processing large contexts. Our approach consistently shows the lowest values across all output token ranges in both metrics, indicating superior performance in reducing meaningless and repetitive content. This suggests that this compression technique is more effective at maintaining coherent and diverse output than other methods like DistilGPT2, KnGPT2, QuantGPT, LightPAFF, and SparseGPT. The advantage of our is particularly pronounced for longer output sequences, where it maintains a significantly lower rate of meaningless and repetitive tokens compared to other approaches. This implies that the proposed method may be especially well-suited for generating longer, more coherent text passages while minimizing irrelevant or redundant content.

**Table 8:** Inference speedup comparison of our compression approach and baseline models on GPT-2. Our approach achieves the highest compression rate (70%) with the fastest latency (~ 10 ms), and the greatest speedup (2.5x) while delivering the best throughput (3,200 sequences /sec).

Model version	Compression rate %	GPU		Throughput (sequences /sec)
		Latency	Speedup	
GPT2	0%	25 ms	Baseline	1,280
DistilGPT	40%	16 ms	1.6x	2,000
KnGPT2	35%	19 ms	1.3x	~ 1,684
QuantGPT	45%	~ 14 ms	1.7x	~ 2,286
LightPAFF	50%	~ 13 ms	1.9x	~ 2,462
SparseGPT	60%	~ 17 ms	1.4x	~ 1,882
Our approach	70%	~ 10 ms	2.5x	**~ 3,200**

The proposed compression approach preserves essential information by reducing the number of unique parameters, which minimizes model complexity and computational cost. This leads to efficient memory utilization and faster training, improving both stability in learning and convergence speed. While reduced complexity and memory utilization are advantageous, the model’s performance is highly sensitive to the chosen compression rate^15^. During execution, it often becomes necessary to dynamically adjust the compression rate (either increasing or decreasing it) to maintain an effective balance between compression and performance. If the pruning strategy is too aggressive, it can lead to significant degradation in model accuracy and generalization^27^. Moreover, the model faces layer-specific challenges, as certain layers (such as attention layers or feed-forward layers in transformer architecture) may be more sensitive to compression than others, especially depending on the task at hand^48^. Techniques like diagonal preservation or row replication may be effective for some layers, but they do not universally perform well across all tasks or domains^49^. For instance, we observed noticeable differences in performance on tasks like SST-2 (sentiment analysis) and QNLI (question-answering). The pruning method that worked well for SST-2 did not yield the same results for QNLI and other tasks^28^, indicating that different tasks may require different compression strategies tailored to the specific layer or model component.

Table 9 presents a comparison of representative pruning methods applied to large language Transformer models, showcasing metrics such as compression rate and speed-up. For the OPT-175B model, the proposed method achieves a compression rate of 56.7% and a speed-up of 1.61x, surpassing SparseGPT’s 54% compression and 1.54 × speed-up. On LLaMA2-7B, it delivers a 63% compression rate and 1.47 × speed-up, outperforming Sheared LLaMA’s 61.4% compression and LLM Pruner’s 20% compression with 1.18 × speed-up. For TinyLLaMA-1B, it achieves the highest compression rate of 45% and a 1.68 × speed-up, exceeding Sheared LLaMA’s 34% compression and 1.57 × speed-up, as well as LLM Pruner’s 12% compression and 1.35 × speed-up. The results indicate that the proposed approach consistently outperforms other methods in terms of compression rate and speed improvement across various models. While our proposed pruning method demonstrates significant improvements in compression rates and speed-up across multiple open-source LLMs, there are certain limitations to consider. One primary constraint is the resource-intensive nature of evaluating and retraining large-scale models. Larger LLMs, such as Falcon and others of similar scale, require substantial computational resources, storage, and energy. Additionally, retraining these models on large datasets to assess the full potential of pruning techniques demands significant financial investment and can take days or even weeks to complete. Due to these challenges, our evaluations were primarily focused on open-access LLMs, including LLaMA2-7B, OPT-175B, and TinyLLaMA-1B, which still offer valuable insights into the effectiveness of the proposed approach. However, we acknowledge that expanding the scope of our evaluations to include larger and more diverse LLMs would further validate the generalizability of our method.

**Table 9:** Comparison of representative pruning method on large language Transformer.

Source Model	Method	Compression rate	Speed Up
OPT-175B^50^	SparseGPT^23^	54%	1.54x
	Our approach	56.7%	1.61x
LLaMA2-7B^51^	LLM Pruner^14^	20%	1.18x
	Sheared LLama^16^	61.4%	-
	Our approach	63%	1.47x
TinyLLaMa-1B^52^	LLM Pruner^14^	12%	1.35x
	Sheared LLama^16^	34%	1.57x
	Our approach	45%	1.68x

## Conclusions

Our research paper comprehensively evaluated our innovative approach for compressing large language models using 11 publicly available datasets, including various metrics. The results of our approach were promising across different scenarios, achieving an overall model compression of 70% while maintaining nearly the same accuracy. Interestingly, with a moderate compression rate of 5% to 65%, model performance not only remained stable but even improved, highlighting the potential for optimized efficiency. This reduces memory requirements and streamlines predictions, making it suitable for real-time applications while contributing to energy savings. However, it is important to note that the effectiveness of our compression approach is highly sensitive to the chosen compression rate and may vary significantly across different tasks. In our future research, we plan to expand the applicability of our compression approach through experiments with diverse pre-trained models across various domains. We also aim to investigate further the trade-offs associated with compression rates and task-specific sensitivities to refine our methodologies. Furthermore, selecting high-quality data from the training dataset can enable further model compression without compromising performance, optimizing efficiency and accuracy.

## Data Availability

The data used in the publication is already openly available. WikiText-2 and WikiText-103 are available at: http://arxiv.org/abs/1609.07843. Penn Treebank (PTB) is available at: https://doi.org/10.1109/SLT.2012.6424228. The General Language Understanding Evaluation (GLUE) benchmark is available at: https://doi.org/10.18653/v1/W18-5446. Contributions to the study of SMS spam filtering: new collection and results available at: https://doi.org/10.1145/2034691.2034742.
